# Explaining Societal Shifts in Victim Blaming and Perpetrator Culpability for Sexual Violence: Evidence From the #MeToo Era

**DOI:** 10.1007/s11199-025-01590-6

**Published:** 2025-07-07

**Authors:** Zoe Abrams

**Affiliations:** Department of Sociology, University of Oxford, 42 Park End St, Oxford, OX1 1 JD England

**Keywords:** Sexual violence, Attitude change, Gender roles, Victim blaming, Kitagawa-Oaxaca-Blinder decomposition, Scotland

## Abstract

Despite increasing public condemnation of sexual violence, it is unclear how these social and political changes correspond to shifts in individual attitudes. This study investigates how attitudes toward sexual violence changed in Scotland, in the context of the #MeToo movement. This study applies the threefold Kitagawa-Oaxaca-Blinder decomposition to the cross-sectional 2014 and 2019 Scottish Social Attitudes Surveys (*N* = 2133) to investigate how attitudes towards victim blaming and perpetrator culpability have changed over time. Perceptions of perpetrator culpability increased and victim blaming decreased significantly between 2014 and 2019. Reduction in victim blaming was associated with the liberalization of gender role attitudes and the increased influence of political interest on attitudes in 2019. Heightened perceptions of perpetrator culpability were associated with declining levels of authoritarianism, as well as greater attitudinal changes amongst women and left-wingers relative to other social groups. The findings highlight the importance of both societal shifts in value orientations and gender attitudes, as well as divergent patterns of change amongst different social groups. Implications for other populations, policy, and consent education are discussed.

**Supplementary Information:**

The online version contains supplementary material available at 10.1007/s11199-025-01590-6.

In 2024, Dominique Pélicot and 50 co-defendants were convicted in France’s largest ever rape trial. Pélicot repeatedly drugged and raped his wife Gisèle without her knowledge over a period of 9 years, inviting over 70 other men to participate. During the widely publicized trial, the defendants frequently cited misconceptions about consent to minimize their personal responsibility (Chrisafis, 2024b). These misconceptions included beliefs such as a woman’s husband being able to consent on her behalf, that the men could not control themselves due to excessive testosterone, or that they “weren’t the type” to commit rape (Chrisafis, 2024a; Willsher, 2024). In the February 2020 trial of media mogul Harvey Weinstein on multiple counts of sexual assault, his accusers were portrayed as lying to promote their careers or as provoking Weinstein’s advances by flirting, attending parties, and accepting invitations to work meetings at hotels (Francescani, 2020).

Sexual violence is a significant social problem. ONS data suggests that nearly 1 in 4 adult women in the United Kingdom have been sexually assaulted since the age of 16 (Rape Crisis, 2024; Topping, 2021). Despite its prevalence, understandings of sexual violence vary across social and legal contexts, influencing both perpetration and reporting (Bedera & Haltom, 2019; Egan & Wilson, 2012; Hahn et al., 2020; Mouilso & Calhoun, 2012; Trottier et al., 2019). Problematic attitudes toward consent and sexuality are a central enabler of violence against women. The examples above demonstrate how attitudes that minimize perpetrators’ culpability for rape and blame the victim contribute to the perpetration and legitimation of sexual violence. The present study examines changes in perceptions of perpetrator culpability and victim blaming between 2014 and 2019 in Scotland, providing insights into shifts in attitudes that are critical to sexual violence.

## Background

### Measuring Attitudes Toward Sexual Violence

Attitudes towards sexual violence are typically measured using individuals’ agreement with ‘rape myths’ – beliefs skeptical of rape allegations, minimizing perpetrators’ culpability, and blaming victims (Burt, 1980). Rape myths provide schemas through which people interpret instances of sexual violence (Abbey & Harnish, 1995; Bohner et al., 2009; Powell & Webster, 2018). The attitudes explored in this study – victim blaming and perceptions of perpetrators’ culpability – reflect key dimensions of rape myths (Johnson et al., 1997; Payne et al., 1999), shedding light on how attitudes toward sexual violence have evolved over time.

A small number of studies conducted amongst U.S. college undergraduates indicate that levels of rape myth acceptance have declined over time (Beshers & DiVita, 2021; Byrne et al., 2021). Yet Beshers and DiVita (2021) found no changes between 2010 and 2017 in the assumption that rape results from men’s need for sex, and Byrne and colleagues (2021) observed that between 1998 and 2018, attitudes to victims’ culpability and credibility were more stable than other rape myths. Both studies used small samples of college undergraduates, limiting their representativeness. The present research examines whether attitudes towards sexual violence in Scotland follow a similar trend, employing a more rigorous sample representative of the Scottish population, and covering a critical (albeit shorter) period in which issues of sexual violence were highly salient.

### The Scottish Context: Policy and Data Landscape

Scotland is a particularly relevant social context for investigating changes in attitudes towards sexual violence, due to its proactive policy intervention and public debate about violence against women led by feminist organizations like Scottish Women’s Aid. These influences have cultivated a rich discursive environment for the formation of attitudes about sexual violence. Since devolution in 1998, Scottish policy has incorporated feminist perspectives that link domestic and sexual violence to patriarchy and misogyny (Charles & Mackay, 2013). Legal reforms have further challenged rape myths by broadening the definition of rape and redefining consent as "free agreement" rather than the absence of resistance (Brindley & Burman, 2012).

The present study employs the Scottish Social Attitudes Survey (SSAS) – a cross-sectional survey of public opinion in Scotland – to measures attitudes towards sexual violence (ScotCen Social Research, 2016, 2023). Beyond the SSAS, an extensive search of the literature highlights a dearth of population-level studies offering panel or repeated cross-sectional data on attitudes toward sexual violence. Although the Australian National Community Attitudes towards Violence against Women Survey (NCAS) provides valuable insights into attitudes toward violence against women, it is not openly accessible and is therefore of limited utility to researchers (Coumarelos et al., 2023). The 2020–24 waves of the American National Election Studies (ANES) included questions on respondents’ perceptions of public responses to sexual harassment, but did not address rape myth acceptance or understandings of what constitutes sexual harassment (American National Election Studies, 2021, 2025).

Amidst this limited data landscape, the SSAS offers the unique benefits of accessible, population-representative data, and detailed questions exploring attitudes toward sexual violence. Questions on violence against women were first incorporated into the SSAS through the Scottish government’s ‘Equally Safe Strategy,’ which was established in June 2014 to tackle violence against women (Reid et al., 2020). The Equally Safe Strategy aims to reduce gender-based violence through legal reforms, funding frontline services for survivors, and supporting educational programs about sexual violence online, in schools, and workplaces (Scottish Government, 2014, 2018).

### The Rise of the #MeToo Movement

A critical influence on attitudes toward sexual violence during the period of study (2014–2019) was the #MeToo movement. Originally founded by activist Tarana Burke, #MeToo gained widespread prominence on Twitter in October 2017 following allegations against Harvey Weinstein, sparking a global shift in discourse that revealed widespread experiences of sexual assault and challenged pre-existing cultures of impunity. The movement was framed as a new form of ‘hashtag feminism,’ in which access to social media lowered the costs of participation in activism, offering newly available tools for mobilization and disclosure experiences of victimization (Clark-Parsons, 2019; Mendes et al., 2018). As a viral phenomenon, the #MeToo movement developed a sustained presence within public discourse – in the year following the movement, the ‘MeToo’ hashtag was used over 19 million times (Anderson & Toor, 2018).

The #MeToo movement was covered widely and favorably in U.K. media – even in the most conservative publications, over 50% of reporting was positive or neutral toward the movement (De Benedictus et al., 2019). In Scotland, #MeToo had a significant political impact, involving widespread media coverage of sexual harassment in the Scottish Parliament. In a case that received substantial attention, the former First Minister of Scotland Alex Salmond was accused of sexual assault by multiple women in the Scottish National Party and civil service, resulting in a trial where he was eventually acquitted (Julios, 2022).

Polling from 2018 indicates significant engagement with the #MeToo movement in the United Kingdom, with 88% of respondents aware of the movement and 60% believing that it increased openness about sexual harassment. In Scotland, these figures were slightly higher at 90% and 65%, respectively. However, Labour and Liberal Democrat voters were more likely to have heard of #MeToo (92% and 96%, respectively) than Conservatives (84%), indicating varying exposure to discourse about sexual violence based on political orientation (YouGov, 2018). Indeed, Google Trends data demonstrates that engagement with issues of sexual violence in Scotland increased over time. Spikes in Google searches for ‘sexual assault’ and ‘sexual harassment’ occurred at the start of the #MeToo movement in November 2017, and another spike in searches for ‘sexual assault’ occurred in January 2019 amidst the arrest of former First Minister of Scotland Alex Salmond on charges of sexual assault (Google Trends, 2024a, 2024b; see Sect. 1 in the online supplement for graphs of these trends). Therefore, by 2019 sexual violence was a more salient and publicly acknowledged issue in Scotland compared with 2014.

### The #MeToo Movement as a Catalyst for Attitude Change

Given its prominence within public discourse, the #MeToo movement may have influenced the formation of attitudes toward sexual violence. Formation is heavily influenced by environmental stimuli, yet scholars disagree regarding how much they are “crystallized in memory, … in-the-moment evaluations, or… hybrid structures” (Albarracin & Shavitt, 2018, p. 301). Some theories portray attitudes as formed in early life and relatively stable ​(Ryder, 1985)​, while established psychological literature on attitude change suggests that attitude formation involves a combination of reference to existing evaluations and the integration of new information (Bohner & Dickel, 2011). Similarly, Kiley and Vaisey (2020) suggest that persistent opinion change is enabled by increased “public salience and social movement activity” about contentious issues (p. 497). Studies using panel data, lab, and field experiments also show that: a) attitude formation can be significantly and enduringly influenced by news media, political campaigns, and social movements, and b) individuals actively seek cues from these stimuli to form opinions (Banaszak & Ondercin, 2016; Broockman & Kalla, 2016; Collingwood et al., 2018; Lenz, 2009; Mutz, 1992). Furthermore, exposure to highly emotive stimuli can reshape individuals’ attitudes (Forgas, 2008; Lambert et al., 2010).

There is considerable evidence that exposure to the #MeToo movement triggered shifts in attitudes toward sexual violence. Small-scale longitudinal studies suggest that people became less likely to dismiss allegations of sexual assault and more willing to engage in collective action against sexual violence after the start of movement (Armstrong & Mahone, 2023; Szekeres et al., 2020). In a U.K. nationally representative survey, 38% of people agreed that their perceptions of acceptable behavior had changed following the #MeToo movement, and 35% stated that they were now more likely to call out sexual harassment (Fawcett Society, 2018). Causal analysis of the impact of the #MeToo movement in 31 OECD countries demonstrates a 10% increase in the reporting of sex crimes to police 6 months after the start of the movement (Levy & Mattsson, 2023). Studies of U.S. college students suggest that people became more likely to label their unwanted sexual experiences as ‘sexual assault’ following the rise of the #MeToo movement (Jaffe et al., 2021; Palmer et al., 2021)​. These studies highlight the potential for social movement activity to shape attitudes about sexual violence. The Scottish Government’s Equally Safe Strategy may have also played a role in attitude change. However, Google Scholar reveals no published evidence of its impact during the study period (2014–2019), and the strategy gained little media or public attention. Indeed, Google Trends data for searches in Scotland show that there were nearly 16 times as many searches for ‘#MeToo’ as for ‘Equally Safe’ (Google Trends, 2025; see online supplement Sect. 1). Therefore, the #MeToo movement may have challenged pre-existing conceptions of sexual violence in Scotland, producing greater rejection of rape myths.

### The Uneven Impact of #MeToo on Attitudes

Both people’s *exposure* to #MeToo discourse and their *reactions* to it are influenced by their attachment to and identification with the issue. Specifically, engagement with the #MeToo movement was greater amongst feminists, women, and those who had experienced sexual violence (Herrera Hernandez & Oswald, 2022; Hoffman, 2021). Similarly, on social media, discussion of sexual violence increased most among groups already engaged with the issue (Baik et al., 2022). Therefore, people who were already more engaged with feminism may have been influenced more strongly by the #MeToo movement than other groups. Meanwhile, people who already exhibited hostile beliefs about sexual violence appeared more resistant to social change. In a small scale U.K. study (*N* = 76), those who denied rape allegations or downplayed sexual aggression were also more likely to express opposition to the #MeToo movement (White et al., 2024). Therefore, #MeToo’s impact on social attitudes may have been greater amongst groups who already had an affinity toward the movement.

Experimental studies from the United States suggest that reactions to #MeToo were also divided along ideological and gendered lines: attitudes toward sexual assault allegations following MeToo varied based on conservative-liberal and Republican-Democrat identities (Hansen & Dolan, 2023; Lisnek et al., 2022). Men expressed greater opposition to the movement than women due to their higher levels of hostile sexism and fears of false allegations (Kunst et al., 2018; Nutbeam & Mereish, 2022). In focus groups with U.K. university students after #MeToo, men exhibited limited engagement with feminist discourses, attempting to distance themselves from perpetrators while also portraying male aggression as biologically inevitable (Burrell, 2022). In anti-feminist online spaces, backlash against the #MeToo movement portrayed women’s allegations as a vindictive strategy to ‘destroy’ and ‘feminize’ men (Dickel & Evolvi, 2022; Maricourt & Burrell, 2022). Just as social media was used to advance feminist discourse via the #MeToo movement, oppositional hashtags like #HimToo and #NotAllMen positioned men victims as of false accusations and feminist overreach (Dejmanee et al., 2020; Flood, 2019).

Taken together, the literature suggests that women, those who are more left-wing, and people with higher levels of political interest may exhibit a greater transformation in their attitudes. However, much of the research cited above comprises small-scale studies without nationally representative samples and does not compare rape myth acceptance before and after the #MeToo movement. The present study benefits from having larger representative samples to track variation in attitudes before and after the social movement began.

### Social and Demographic Influences on Attitudes Toward Sexual Violence

The use of nationally representative samples is essential for understanding changes in attitudes toward sexual violence, because rape myth acceptance varies across social demographics. Certain groups including men, more religious individuals, and those with lower education and socioeconomic status, exhibit higher levels of rape myth acceptance (Barnett et al., 2018; Nagel et al., 2005; Powers et al., 2015; Prina & Schatz-Stevens, 2020; Sierra et al., 2010). Additionally, cohort socialization and maturation over the life course mean that age has a non-linear effect, with both younger and older age groups exhibiting greater rape myth acceptance (Flood & Pease, 2009; Johnson et al., 1997; Suarez & Gadalla, 2010). Analysis of attitudes in deprived areas of Scotland suggests divergence between men and women’s perspectives: women emphasized the importance of mutual and respectful sexual relationships amidst frequent experiences of sexual coercion, whereas men exhibited greater victim blaming and limited understandings of consent (McDaid et al., 2019). Thus, changes in attitudes towards sexual violence may vary across social groups within Scottish society.

### Ideologies and Gender Norms

Differences in rape myth acceptance across social groups are shaped by the dominant value orientations, and social and political ideologies within those groups (PettyJohn et al., 2023). Analysis conducted using a convenience sample of Scottish students suggests that individuals with greater right-wing authoritarianism and sexism endorsed rape myths more strongly, particularly the notion that women lie about rape or that perpetrators do not intend to rape (Manoussaki & Veitch, 2015). Conservatism and system justifying beliefs are associated with greater rape myth acceptance (Martini & De Piccoli, 2020). Hostile sexism – overt prejudice towards women – also facilitates rape myth acceptance by portraying women as fickle and manipulative (Duran et al., 2018). These beliefs facilitate the denial of rape allegations and the minimization of perpetrators’ malicious intent by portraying non-consent as ‘token resistance’ to intercourse and allegations of sexual assault as strategic attempts to undermine men’s authority (Abrams et al., 2003; Lys et al., 2021; Rollero & Tartaglia, 2018).

Rape myth acceptance is also associated with support for biological gender essentialism (the belief that gender differences are rooted in innate biological differences rather than socialization) and traditional gender roles (Lutz-Zois et al., 2015). These roles are reinforced through benevolent sexism, which presents women as inferior but requiring male protection, and makes women’s value conditional on their fulfilment of traditional femininity (Glick & Fiske, 1997; Gothreau et al., 2022)​. Therefore, it is associated with greater victim blaming in rape cases where victims have violated traditional roles by wearing revealing clothes, drinking, and pursuing relationships with men (Chapleau et al., 2007; Koepke et al., 2014; Viki & Abrams, 2002). Conversely, male perpetrators’ actions are more often excused when portrayed as enacting traditional masculine roles of protectiveness and leadership (Agadullina et al., 2022). These traditional gender roles normalize practices of female submission and male sexual aggression, reducing perceptions of perpetrator culpability (Duran et al., 2011; Fraser, 2015).

### Societal Liberalization

Nonetheless, processes of social change occurring since the 1970s have facilitated a decline in support for traditional gender roles in the United States and Europe. These norms have increasingly been replaced by diverse forms of egalitarianism that emphasize individual rights, self-expression and hence women’s ability to choose a variety of social roles (Bornatici et al., 2020; Brooks & Bolzendahl, 2004; Cotter et al., 2011; Inglehart & Norris, 2003; Knight & Brinton, 2017; Park et al., 2013). The global diffusion of policies and legal frameworks that emphasize women’s autonomy is associated with more egalitarian gender role attitudes and with greater opposition to violence against women (Frank et al., 2010; Pandian, 2019; Pierotti, 2013). Therefore, it is plausible that changes in traditional norms may have also influenced attitudes toward sexual violence in Scotland.

Long term social attitude trends documented by the British Social Attitudes survey suggest a liberalization of beliefs about gender and sexuality across the United Kingdom. Since 1983, acceptance of abortion increased by 39%, premarital sex by 36%, and homosexuality by 50% (Clery, 2023). In contrast, support for traditional gender roles decreased by 39% (Allen & Stevenson, 2023; Clery, 2023). Much of this societal liberalization has occurred within the past 15 years – there was minimal change in levels of authoritarianism between 1986–2011, compared with a 12.2% decrease in support for authoritarian values between 2011–21 (Curtice, 2023). Thus, given the established links between rape myth acceptance and support for traditional gender roles, authority, and social hierarchy (Lutz-Zois et al., 2015; Martini & De Piccoli, 2020; PettyJohn et al., 2023), this observed process of societal liberalization may enable changes in understandings of sexual violence.

### Summary and Study Aims

The literature reviewed in this section suggests two processes that may produce changes in attitudes toward sexual violence: a) liberalization of societal attitudes, and b) group-based exposure to and mobilization by the #MeToo movement. The studies outlined above suggest that acceptance of rape myths is predicted by traditionalism and authoritarianism, but that public support for these values is declining. Consequently, shifts in beliefs about sexual violence may be influenced by processes of social change that challenge the ideological frameworks that enable rape myth acceptance. Existing studies also demonstrate that the #MeToo movement had widespread but uneven effects on attitudes towards sexual violence, with participation in and support for the movement being concentrated among groups already predisposed to engage with feminist discourse, while others showed backlash towards the movement. However, there has been little exploration of how these processes may be reflected in population-level patterns of attitude change. The present study addresses this gap by examining whether observed changes in attitudes toward sexual violence between 2014 and 2019 are consistent with expectations derived from these two pathways of change: social attitude liberalization and group-based engagement with the #MeToo movement.

## Current Study

The present study investigates differences in Scottish attitudes towards sexual violence between 2014 and 2019, and the main factors that explain the variation in attitudes between these two years. The study examines beliefs that reflect two key dimensions of rape myths (Manoussaki & Veitch, 2015; Payne et al., 1999). These include the denial that the perpetrator committed violence (*Perpetrator Culpability*), and the transfer of responsibility for the perpetrator’s actions onto the victim (*Victim Blaming*).

This study addresses the following questions:

1. Has there been an increase in perceptions of perpetrators’ culpability for rape and a decrease in victim blaming between 2014 and 2019?
2. To what extent are shifts in attitudes towards sexual violence explained by changes in samples’ characteristics, or the changing effects of those characteristics between 2014 and 2019?

It will investigate the following hypotheses:

- Hypothesis 1: Victim blaming would decrease, and perceptions of perpetrators’ culpability would increase between 2014 and 2019.
- Hypothesis 2: The liberalization of social attitudes — specifically political orientation and gender role attitudes — accounts for a significant proportion of the change in attitudes towards sexual violence between the 2014 and 2019 samples.
- Hypothesis 3a: A significant proportion of the change in attitudes towards sexual violence is explained by the differing effects of predictor variables in 2014 compared to 2019.
- Hypothesis 3b: Predictors that may reflect greater exposure to and identification with #MeToo will have stronger effects in 2019 than in 2014. This will explain a proportion of the change in attitudes towards sexual violence. These include:
- A stronger association between being a woman and lower victim blaming/higher perceptions of perpetrator culpability,
- A stronger association between higher political interest and lower victim blaming/higher perceptions of perpetrator culpability,
- A stronger association between more left-wing/libertarian political orientation and lower victim blaming/higher perceptions of perpetrator culpability.

To address these questions, this study employs the Kitagawa-Oaxaca-Blinder (KOB) decomposition (Blinder, 1973; Kitagawa, 1955; Oaxaca, 1973). This method is used to quantify the extent to which differences in attitudes toward sexual violence between 2014 and 2019 are explained by differences in the samples’ *characteristics* (known as ‘endowment effects’) and differences in the *effects* of the samples’ characteristics (‘coefficient effects’). Although KOB decomposition was originally designed to investigate wage discrimination—for example, by decomposing groups’ wage differences into the proportion attributable to variation in educational qualifications versus to the differential rewarding of those qualifications (Blau & Kahn, 2017; Jones et al., 2018; Kim, 2010)—it has recently been used to measure a wider range of discrepancies in outcomes between groups, including attitudinal differences (Beja, 2018; Borwein et al., 2024; Etezady et al., 2021; Zegeye et al., 2021).

The KOB decomposition is particularly well-suited for understanding changes in attitudes towards sexual violence, as it gives insight into the plausibility of the two mechanisms of attitude change outlined above: a) broad liberalization of societal attitudes, and b) group-based exposure to and mobilization by the #MeToo movement. The KOB decomposition quantifies the proportion of attitude change that is explained by the impact of differences in the 2014 and 2019 samples’ *characteristics* (endowment effects), illustrating the possible role of societal liberalization in shifts in attitudes toward sexual violence (Hypothesis 2). Differences in the *effects* of the samples’ characteristics (coefficient effects) may indicate the mobilization of specific groups – for example, women, liberals, left-wingers, and people who participate heavily in politics—around issues of sexual violence amidst the #MeToo movement (Hypotheses 3a-3biii). While this study does not have the scope to directly test the causal factors that drive these coefficient effects, the KOB decomposition offers some indication of how the changing social and political context might contribute to differences in attitudes between 2014 and 2019.

To the authors’ knowledge, the present study is the first to use the SSAS to analyze predictors of attitudes toward sexual violence. ScotCen Social Research has conducted some basic descriptive comparison of attitudes using the 2014 and 2019 SSAS but did not evaluate how key predictors of attitudes toward sexual violence varied across years (Reid et al., 2020).

The present study bridges gaps in the literature by combining sociological and psychological perspectives to explore how demographic and attitudinal factors shape attitudes toward sexual violence. While the literature reviewed above provides important insights into individuals’ attitudes toward rape, there are few large-scale studies exploring how attitudes have changed over time. This question is especially important for the study period (2014–19), during which the issue of sexual violence gained political salience through the #MeToo movement.

## Method

### Data

The 2014 and 2019 SSAS are two cross-sectional surveys measuring attitudes towards a range of topics within Scotland. To minimize social desirability bias, questions about violence against women were answered in a self-completion section, while the rest were administered by an interviewer (Reid et al., 2020). After exclusions, the final sample size consisted of 2133 respondents, representing 84.9% of the merged dataset’s original total (see Section X of the online supplement for further details). Of this sample, 1276 respondents were from 2014, and 857 were from 2019. Missing data analysis indicated that data were missing at random. Descriptive statistics (displayed in Table 1) show that the 2014 and 2019 samples have broadly comparable demographic characteristics.

**Table 1:** Descriptive Statistics: Scottish Social Attitudes Survey, 2014 and 2019 (*N* = 2133)

		Frequency							
		Unweighted				Weighted			
Variable	Value	2014* N*	2014*%*	2019* N*	2019*%*	2014* N*	2014*%*	2019* N*	2019*%*
Gender	Man	654	43.7%	468	46.2%	718	47.9%	480	47.8%
	Woman	844	56.3%	545	53.8%	780	52.1%	524	52.2%
Race	White	1440	96.3%	974	96.53%	1403	93.6%	942	93.9%
	Non-White	55	3.7%	35	3.47%	94	6.3%	62	5.9%
Age	18–24	100	6.68%	45	4.44%	175	11.7%	88	8.8%
	25–34	194	13%	130	12.8%	244	16.3%	195	19.4%
	35–44	251	16.8%	130	12.8%	241	16.1%	151	15.0%
	35–54	259	17.3%	183	18.1%	280	18.7%	176	17.6%
	55–64	264	17.6%	186	18.4%	232	15.5%	164	16.3%
	65 +	430	28.7%	339	33.5%	327	21.8%	230	22.9%
Class (occupational category)	Routine	178	12.9%	127	13.4%	173	11.5%	129	12.9%
	Semi-routine	261	18.9%	163	17.2%	266	17.7%	157	15.6%
	Lower supervisory & technical	134	9.68%	97	10.2%	122	8.1%	93	9.3%
	Small employers & own account workers	123	8.89%	89	9.32%	108	7.2%	87	8.6%
	Intermediate occupations	204	14.7%	97	10.2%	202	13.5%	82	8.2%
	Lower professional & managerial; higher technical & supervisory	321	23.2%	262	27.6%	168	11.2%	252	25.1%
	Employers in large organizations; higher managerial & professional	163	11.8%	115	12.1%	151	10.0%	120	8.3%
Education	None	309	20.7%	187	18.7%	269	17.9%	155	15.4%
	Standard Gd/GCSE	357	24%	226	22.6%	344	22.9%	213	21.3%
	Highers/A-Levels	274	18.4%	149	14.9%	299	20.0%	160	15.9%
	Degree/HE	550	36.9%	438	43.8%	577	38.5%	465	46.4%
Marital status	Not married	710	47.4%	474	46.8%	610	40.7%	384	38.3%
	Married	788	52.6%	538	53.2%	889	59.3%	619	61.7%
Religious identity	Not religious	637	42.7%	621	64.8%	658	43.9%	640	63.7%
	Religious	855	57.3%	338	35.2%	836	55.8%	364	36.3%

### Dependent Variables

Confirmatory factor analysis using the lavaan R package (Roseel, 2012) confirmed that the two dependent variables—Victim Blaming and Perpetrator Culpability—are distinct constructs. The model fit was excellent (RMSEA = 0.008, CFI = 1.000), with all factor loadings highly significant (full results reported in Sect. 2.1 of the online supplement). The significant covariance (−0.50***) between the factors indicates that lower perceptions of Perpetrator Culpability are associated with greater Victim Blaming.

Multiple-group confirmatory factor analysis was conducted to test measurement invariance of the dependent variables across sexes and survey years. This analysis ensured that constructs were measured equivalently despite the potential for changing understandings of sexual violence over time and differing reactions to questions based on sex. Both the configural and metric invariance models were an excellent fit (RMSEA = 0.000, CFI = 1.000), and the strict invariance model, which constrains factor loadings, intercepts, and residual variances to be equal, also fits well (RMSEA = 0.035, CFI = 0.989). These model fit indices suggest that differences between groups are unlikely due to measurement error (Hirschfeld & von Brachel, 2014), ensuring that the dependent variables accurately measure Victim Blaming and perceptions of Perpetrator Culpability across survey years and sexes.

#### Perceived Perpetrator Culpability

To measure perceived Perpetrator Culpability, a variable was created that averaged scores of two questions relating to false rape accusations and male sexuality. Respondents were asked to indicate their level of agreement with two statements: “*Women often lie about being raped*” (*M* = 3.37, *SD* = 1.04) and “*Rape results from men being unable to control their need for sex*” (*M* = 3.12, *SD* = 1.33). Both questions were measured on a 5-point scale, with higher scores representing greater opposition to the statements.

#### Victim Blaming

To measure Victim Blaming, a variable was created that averaged scores from two questions evaluating the blame attributable to a woman who had engaged in behavior that violated traditional gender norms. Respondents were asked how much a woman is to blame for being raped if she a) “*wears very revealing clothing on a night out*” (*M* = 2.17, *SD* = 1.82), and b) “*is very drunk*” (*M* = 2.12, *SD* = 1.83). Each of these questions was measured on a 7-point scale, where lower scores represent less blame attributed to the woman.

### Covariates

Because the K–O-B decomposition of categorical variables depends on the choice of omitted baseline group (Oaxaca & Ransom, 1999), the standard practice of converting categorical variables into scale or dummy variables was followed (Jann, 2008). See Sect. 2.3 of the online supplement for an explanation of how this choice was determined. For consistency, these variables were also used in the hierarchical regression analyses.

#### Demographic Variables

Gender was converted from a two-group categorical variable into a dummy indicator for being a woman, in which 0 = *man* and 1 = *woman*. Religious identity was collapsed into a dummy variable where 0 = *not religious*, and 1 = *religious*. Marital status was collapsed into a dummy variable where 0 = *not married*, and 1 = *married*. Class was measured with occupational categories on a 1–7 scale, in which 1 referred to routine occupations, and 7 referred to professional occupations. Education levels were measured using a 4-point scale, where 1 = ‘*none*,’ and 4 = ‘*Degree/HE*.’

#### Behavioral and Attitudinal Variables

Several attitudinal and behavioral variables were included in the analysis to test H2, H3a, and H3bi-iii. *Political Interest* was measured using a 5-point scale variable measuring self-reported levels of political interest, where 1 = ‘*none at all*’ and 5 = ‘*a great deal*. The effects of online news engagement on attitudes were also tested in preliminary analyses. However, there were over 300 missing cases for this variable in the 2019 sample, and it was not significantly associated with either dependent variable. Therefore, it was excluded from the main analyses. *Gender Role Attitudes* were measured by averaging the scores from two vignette questions asking about the respondent’s hypothetical response to a child asking for a non-gender stereotypical toy in a shop (one question where the child was a boy, and another where the child was a girl). This variable is measured on a 3-point scale, where higher scores represent support for more traditional gender roles. These two variables were highly correlated with each other and had good internal consistency (Cronbach’s alpha = .86). *Left–Right Political Orientation* was measured using an average score (scaled from 1–5) provided by the survey, derived from items measuring attitudes towards economic inequality and fairness. *Libertarian-Authoritarian Political Orientation* was measured using an average score (scaled from 1–5) provided by the survey, derived from items measuring attitudes towards traditional values, law and order, free speech, and obedience to authority. On these scales, a higher score indicates more right-wing/authoritarian political attitudes. See Sect. 2.2 of the online supplement for further details.

## Results

### Mean Differences in Attitudes Between 2014–2019

To understand changes in the political and social landscape between 2014 and 2019, two-tailed *t*-tests were performed on the attitudinal and behavioral variables. There were significant differences in Political Interest, Gender Role Attitudes, and Libertarian-Authoritarian and Left–Right Political Orientation scores. The 2019 sample was more libertarian, left-wing, and politically engaged, as displayed in Table 2. There was a particularly large shift away from traditional gender roles (−8.03%). These changes are relevant for subsequent testing of H2.

**Table 2:** Two-Tailed T-Tests for Attitudinal and Behavioral Variables

Attitude	Year	*df*	*M*	*SD*	Difference in Means	*p*
Gender Role Attitudes	2014	2150	1.73	0.72	0.24	
	2019		1.49	0.65	(−8.03%)	< .001
Left–Right Orientation	2014	1937	2.36	0.75	0.12	
	2019		2.24	0.80	(−2.34%)	.001
Libertarianism-Authoritarianism Orientation	2014	1788	3.60	0.70	0.17	
	2019		3.42	0.83	(−3.44%)	< .001
Political Interest	2014	2153	3.00	1.19	−0.15 (2.90%)	.003
	2019		3.15	1.21		

To test H1, two-tailed *t*-tests compared differences in Victim Blaming and perceived Perpetrator Culpability between 2014 and 2019. As hypothesized, there were significant reductions in Victim Blaming (3.44% lower in 2019) and increases in perceptions of Perpetrator Culpability (9.06% higher in 2019), as shown in Table 3.

**Table 3:** Two-Tailed T-Tests for Victim Blaming and Perpetrator Culpability

Attitude	Year	*df*	*M*	*SD*	Difference in Means	*p*
Victim Blaming	2014	2082	2.25	1.73		
	2019		2.01	1.62	−0.24 (−3.44%)	< .001
Perpetrator Culpability	2014	1910	3.06	0.89		
	2019		3.51	0.95	0.45 (9.06%)	< .001

### Assessment of the Role of Demographic and Attitudinal Variables in 2014 Versus 2019

Prior to conducting the KOB decomposition analysis, it is necessary to establish the key predictors of Victim Blaming and perceptions of Perpetrator Culpability, and to explore whether these changed between 2014 and 2019. Hierarchical regression analyses were conducted for each dependent variable to establish the key predictors. In the first model, the independent variables were demographic characteristics (sex, age and age squared, marital status, class, education, religious identity). A quadratic term was included for age, to account for the possibility that it has a non-linear effect on attitudes. The next model added levels of Political Interest. The final model also incorporated attitudinal variables, including Gender Role Attitudes, Libertarianism-Authoritarianism, and Left–Right Political Orientation score. Tables 4 and 5 report unstandardized coefficients, while standardized coefficients are displayed in Figs. 2–3 to demonstrate the relative effect size of each predictor in 2014 and 2019 for each dependent variable.

**Table 4:** Hierarchical Regression Analyses – Victim Blaming

	**2014**		**2014**		**2014**		**2019**		**2019**		**2019**	
*Predictors*	*Estimates*	*SE*	*Estimates*	*SE*	*Estimates*	*SE*	*Estimates*	*SE*	*Estimates*	*SE*	*Estimates*	*SE*
Intercept	3.42***	(0.40)	3.48***	(0.40)	1.34**	(0.51)	3.50***	(0.46)	3.65***	(0.46)	2.06***	(0.57)
Gender (Woman)	0.09	(0.09)	0.07	(0.09)	0.19*	(0.09)	−0.05	(0.10)	−0.08	(0.10)	0.07	(0.10)
Age squared	0.001***	(< 0.001)	0.001***	(< 0.001)	0.001***	(< 0.001)	0.001***	(< 0.001)	0.001***	(< 0.001)	0.001***	(< 0.001)
Age	−0.08***	(0.02)	−0.08***	(0.02)	−0.08***	(0.02)	−0.09***	(0.02)	−0.09***	(0.02)	−0.09***	(0.02)
Religious identity (Religious)	0.26**	(0.09)	0.27**	(0.09)	0.14	(0.09)	0.39***	(0.11)	0.38***	(0.11)	0.27*	(0.11)
Education	−0.07	(0.05)	−0.06	(0.05)	−0.003	(0.05)	−0.05	(0.05)	0.01	(0.05)	0.04	(0.05)
Class	−0.03	(0.03)	−0.03	(0.03)	−0.02	(0.02)	−0.02	(0.03)	−0.004	(0.03)	−0.001	(0.03)
Marital status (Married)	0.06	(0.09)	0.06	(0.09)	0.03	(0.09)	−0.06	(0.10)	−0.02	(0.10)	−0.02	(0.10)
Political interest			−0.05	(0.04)	0.01	(0.04)			−0.16***	(0.05)	−0.11*	(0.05)
Libertarian-Authoritarian					0.22**	(0.07)					0.13	(0.07)
Left–Right					0.13*	(0.06)					0.12	(0.06)
Gender roles					0.35***	(0.07)					0.40***	(0.08)
Observations	1285		1285		1276		867		867		860	
*R*^*2*^ / *R*^*2*^ adjusted	0.15 / 0.15		0.15 / 0.15		0.19 / 0.18		0.17/ 0.16		0.18 / 0.18		0.22 / 0.21	

*Note*. Significance codes: *** *p* < .001, ** *p* < .01, * *p* < .05

**Table 5:** Hierarchical Regression Analyses – Perpetrator Culpability

	**2014**		**2014**		**2014**		**2019**		**2019**		**2019**	
*Predictors*	*Estimates*	*SE*	*Estimates*	*SE*	*Estimates*	*SE*	*Estimates*	*SE*	*Estimates*	*SE*	*Estimates*	*SE*
Intercept	3.01***	(0.22)	2.92***	(0.22)	4.35***	(0.28)	2.81***	(0.28)	2.73***	(0.28)	4.47***	(0.34)
Gender (Woman)	0.05	(0.05)	0.08	(0.05)	0.04	(0.05)	0.26***	(0.06)	0.28***	(0.06)	0.20***	(0.06)
Age Squared	< 0.001	(< 0.001)	< 0.001	(< 0.001)	< 0.001	(< 0.001)	< 0.001	(< 0.001)	< 0.001	(< 0.001)	< 0.001*	(< 0.001)
Age	−0.002	(0.01)	−0.004	(0.01)	−0.004	(0.01)	0.01	(0.01)	0.01	(0.01)	0.01	(0.01)
Religious Identity (Religious)	−0.12*	(0.05)	−0.14**	(0.05)	−0.08	(0.05)	−0.10	(0.07)	−0.09	(0.07)	−0.03	(0.06)
Education	0.09**	(0.02)	0.07**	(0.03)	0.02	(0.03)	0.17***	(0.03)	0.14***	(0.03)	0.07*	(0.03)
Class	0.03*	(0.01)	0.02	(0.01)	0.01	(0.01)	0.01	(0.02)	0.004	(0.02)	−0.003	(0.02)
Marital Status (Married)	0.04	(0.05)	0.04	(0.05)	0.06	(0.05)	0.07	(0.06)	0.05	(0.06)	0.06	(0.06)
Political Interest			0.08***	(0.02)	0.03	(0.02)			0.09***	(0.03)	0.02	(0.03)
Libertarian-Authoritarian					- 0.32***	(0.04)					−0.35***	(0.04)
Left–Right					0.07*	(0.03)					−0.06	(0.04)
Gender Roles					−0.10**	(0.04)					−0.06	(0.05)
Observations	1285		1285		1276		865		865		857	
*R*^*2*^ / *R*^*2*^ adjusted	0.08 / 0.07		0.08 / 0.08		0.15 / 0.15		0.13 / 0.12		0.14 / 0.13		0.22 / 0.21	

*Note*. Significance codes: *** *p* < .001, ** *p* < .01, * *p* < .05

### Victim Blaming

Table 4 shows that in 2014, age had a significant non-linear association with Victim Blaming, decreasing and then increasing around age 38. After controlling for attitudinal variables, women exhibited higher levels of Victim Blaming than men. There was a significant association between Victim Blaming and being more authoritarian, right-wing, and having more traditional gender role attitudes.

In 2019, there was once again a large, significant and non-linear effect of age on Victim Blaming. Unlike in 2014, Political Interest was significant and had a larger effect, with higher levels of interest predicting less Victim Blaming. This result may support H3bii. The association between being a woman and higher levels of Victim Blaming was non-significant in 2019. While there was a significant association between being religious and higher levels of Victim Blaming, the size and significance of this relationship decreased after controlling for attitudinal variables. Like in 2014, having more traditional Gender Role Attitudes was associated with greater Victim Blaming.

**Fig. 1:**
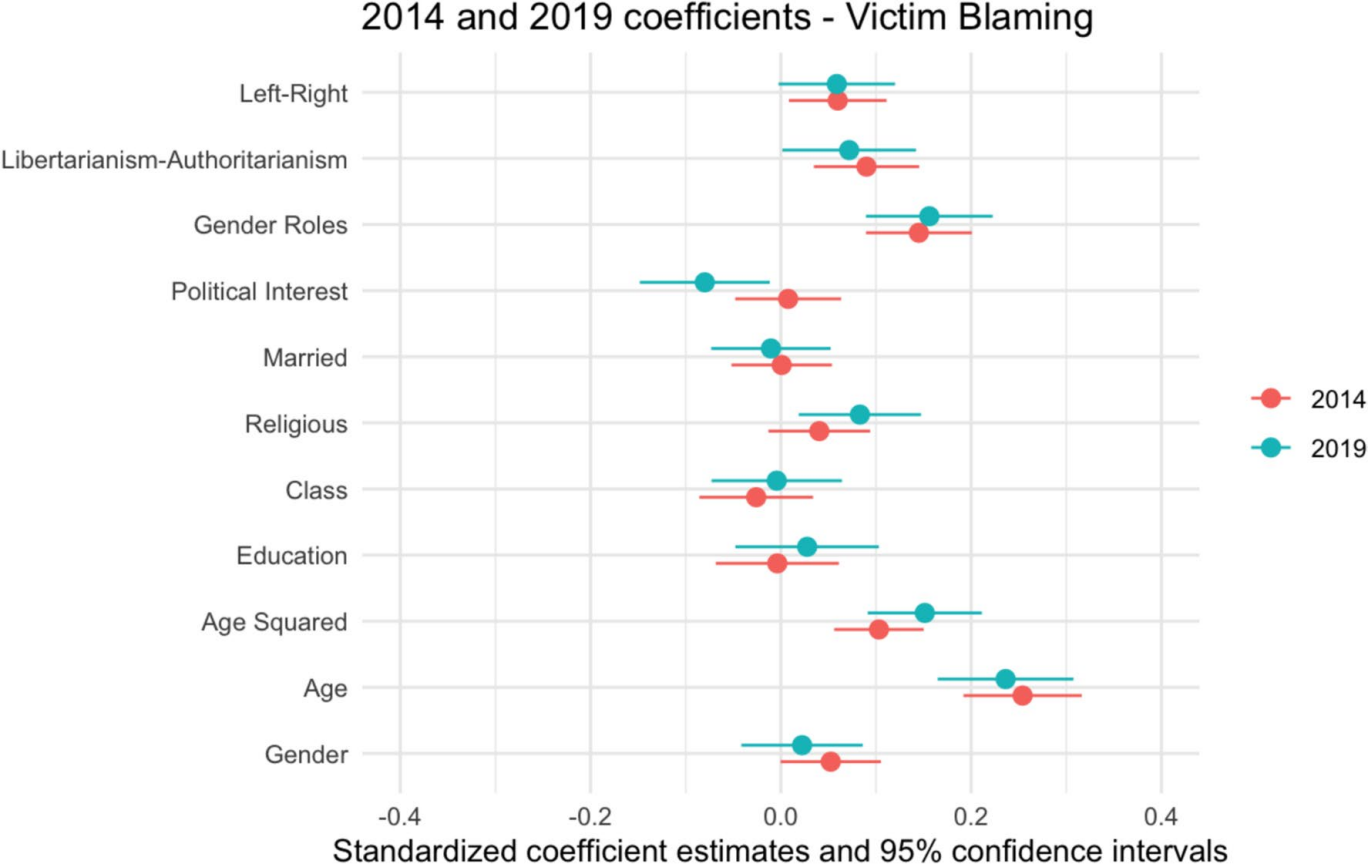
Plotted Standardized Coefficients for Victim Blaming, 2014 and 2019

The results in Table 4 highlight differing drivers of Victim Blaming in 2014 and 2019. The association between Victim Blaming and having a more authoritarian/right-wing Political Orientation was smaller and non-significant in 2019. However, Fig. 1 shows that the association between high levels of Political Interest and lower levels of Victim Blaming was stronger in 2019 than in 2014.

### Perpetrator Culpability

Table 5 shows that in 2014, there was a significant negative relationship between being religious and perceptions of perpetrators’ culpability, whereas having a higher level of education and class status were linked to greater perceptions of culpability. After including behavioral and attitudinal variables, these effects became non-significant. Holding authoritarian political beliefs and traditional gender role attitudes was significantly associated with lower perceptions of Perpetrator Culpability. Surprisingly, there was a significant positive relationship between right-wing orientation and perceptions of Perpetrator Culpability, where right-wingers had higher perceptions of Perpetrator Culpability.

In 2019, having higher levels of education and being a woman were significant predictors of perceptions of Perpetrator Culpability, even after controlling for attitudinal variables. The positive effect of Political Interest became smaller and non-significant after controlling for Political Orientation and Gender Role Attitudes. Of the attitudinal variables, only Libertarianism-Authoritarianism was significant, with more authoritarian individuals having lower perceptions of culpability (consistent with 2014). The effect of Left–Right Political Orientation was non-significant and negative in 2019, implying those on the left had higher perceptions of Perpetrator Culpability.

**Fig. 2:**
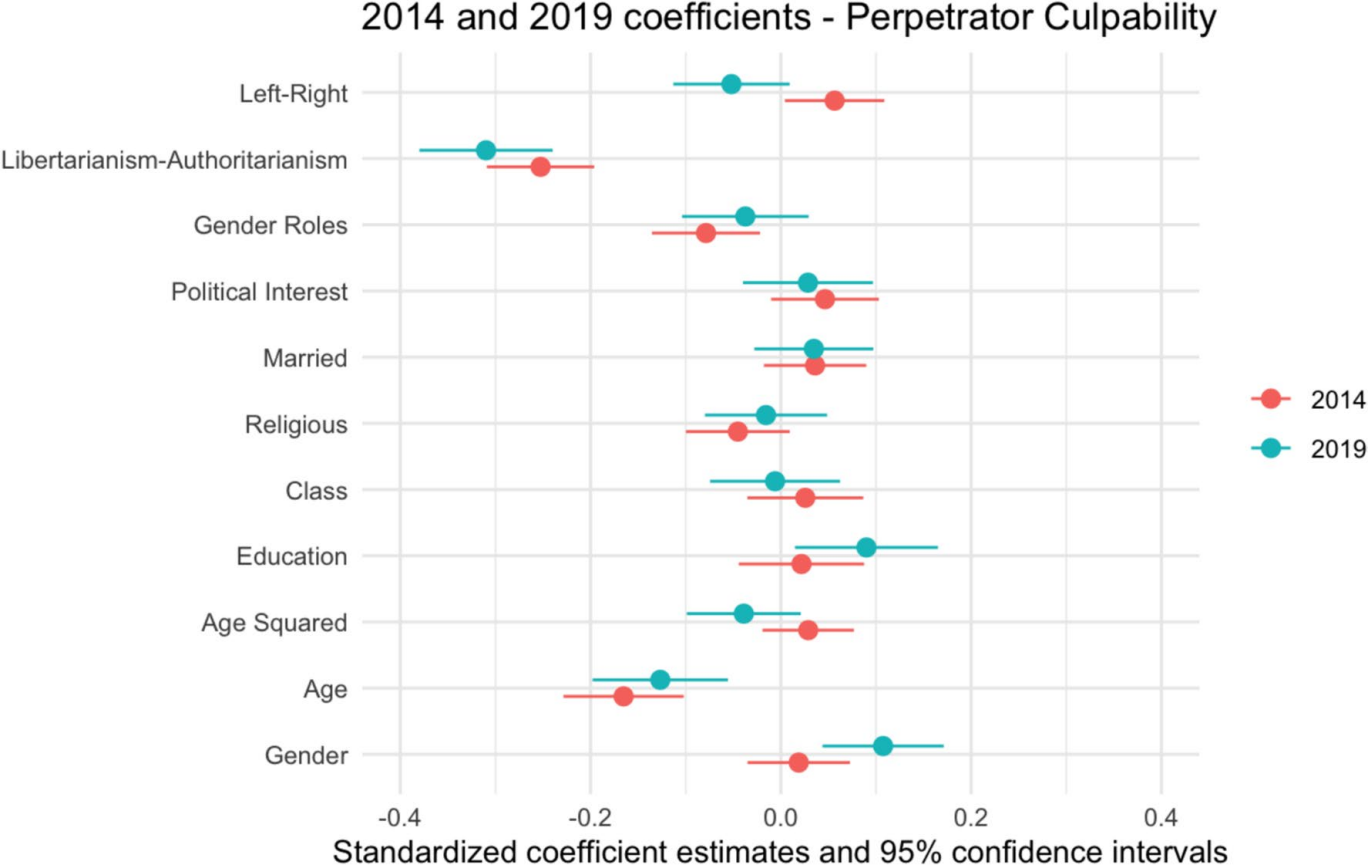
Plotted Standardized Coefficients for Perpetrator Culpability, 2014 and 2019

The analysis presented in Table 5 and the standardized coefficients in Fig. 2 reveal variations in predictors of perceived Perpetrator Culpability between 2014 and 2019. As shown in Fig. 2, the effect of education and of being a woman was larger in 2019, while the direction of the relationship between Left–Right Political Orientation and Perpetrator Culpability changed.

### Summary

The findings outlined above suggest that there are differences in the significant predictors of Victim Blaming and Perpetrator Culpability. Age, religion, and Political Interest predicted Victim Blaming, whereas being a woman, education, and Libertarianism-Authoritarianism had a more significant effect on perceptions of Perpetrator Culpability. Both beliefs were predicted by Gender Role Attitudes. The differing predictors and relationships between years outlined in Tables 4 and 5 help to contextualize the coefficient and endowment effects outlined in the decomposition below.

### Decomposition of Attitudinal Differences Between 2014–2019

Kitagawa-Oaxaca-Blinder (KOB) decomposition was used to investigate different components of the changes in Victim Blaming and Perpetrator Culpability between 2014 and 2019. The decomposition method estimates separate linear regressions for the 2014 and 2019 samples. It then estimates the gap in mean attitudes (Δ $\overline{Y }$) predicted by the linear regressions, and partitions it into distinct components that mathematically explain the gap in attitudes: differences in characteristics (endowments), differences in the effects of the characteristics across groups (coefficients), and the interaction between endowment and coefficient effects (interaction) (Hvalac, 2022; Jann, 2008).

This is represented as:

Equation 1: Threefold Kitagawa-Oaxaca-Blinder decomposition from the perspective of Group B

1 $$\Delta\overline Y=\underbrace{\left({\overline X}_A-{\overline X}_B\right)'{\widehat\beta}_B}_{endowments}+\underbrace{{\overline X}_B'\left({\widehat\beta}_B-{\widehat\beta}_B\right)}_{coefficients}+\underbrace{\left({\overline X}_A-{\overline X}_B\right)'\left({\widehat\beta}_B-{\widehat\beta}_B\right)}_{interaction}$$

In Eq. 1, Group A refers to 2014, and Group B to 2019. The decomposition offers a counterfactual analysis of how mean attitudes towards sexual violence in 2019 would change if respondents in 2019 had the endowments and coefficients of the respondents in 2014, helping to investigate H2–H3biii. Each component is estimated separately, so the endowment effects represent what the change in mean attitudes in 2019 would be if the 2019 respondents *had the same characteristics* as respondents in 2014, while retaining their own coefficients (H2). Meanwhile, the coefficient effects represent what the change in mean attitudes towards sexual violence in 2019 would be if respondents’ characteristics had *the same effects* (coefficients) as in 2014, while retaining their own endowments (H3a–H3biii). This procedure enables the decomposition to distinguish between changes driven by differences in the samples’ characteristics, and changes driven by changes in the *effects* of the samples’ characteristics.

The same model was used for the KOB decomposition as in the regression analyses above. The’oaxaca’ R package (Hvalac, 2022) was used to conduct the analysis. Two threefold decompositions were performed, one for each dependent variable. Standard errors for the decomposition were calculated based on 100 bootstrapping replicates. Both confidence intervals and *p*-values are reported for estimates, with full results shown in Sect. 3.1 and 3.2 of the online supplement.

### Victim Blaming

The KOB decomposition predicts a mean score of 2.21 in 2014 and 1.98 in 2019, with a difference of 0.23. This implies lower levels of Victim Blaming in 2019, supporting H1. As displayed in Table 6, the endowment and coefficient components of the gap contribute to 58.82% and 58.34% of the gap, respectively. The interaction component was negative and contributed to −17.16% of the gap. These findings indicate that the decline in Victim Blaming in 2019 can be explained both by changes in sample characteristics and to the differing effects of their characteristics, supporting H2 and H3a.

**Table 6:** Summary of Threefold Victim Blaming Decomposition by Variable Group (Brackets Representing Percentage of Gap)

Component/Variable Category	Endowment	Coefficient	Interaction	Total Contribution
Overall Decomposition				
Total contribution	0.14 (58.82%)	0.13 (58.34%)	−0.04 (−17.16%)	0.23 (100.00%)
*SE*	0.05	0.06	0.04	
95% CI	(0.04, 0.23)	(0.01, 0.26)	(−0.11, 0.03)	
*p*	.004	.035	.284	
By Variable Group				
Demographic	−0.01	0.22	−0.02	0.18
	(−5.81%)	(95.46%)	(−10.57%)	(79.04%)
Attitudinal/Behavioral	0.15	0.63	−0.02	0.77
	(64.63%)	(276.64%)	(−6.59%)	(334.67%)
Intercept		−0.72 (−313.76%)		−0.72 (−313.76%)

#### Endowment Effects

**Fig. 3:**
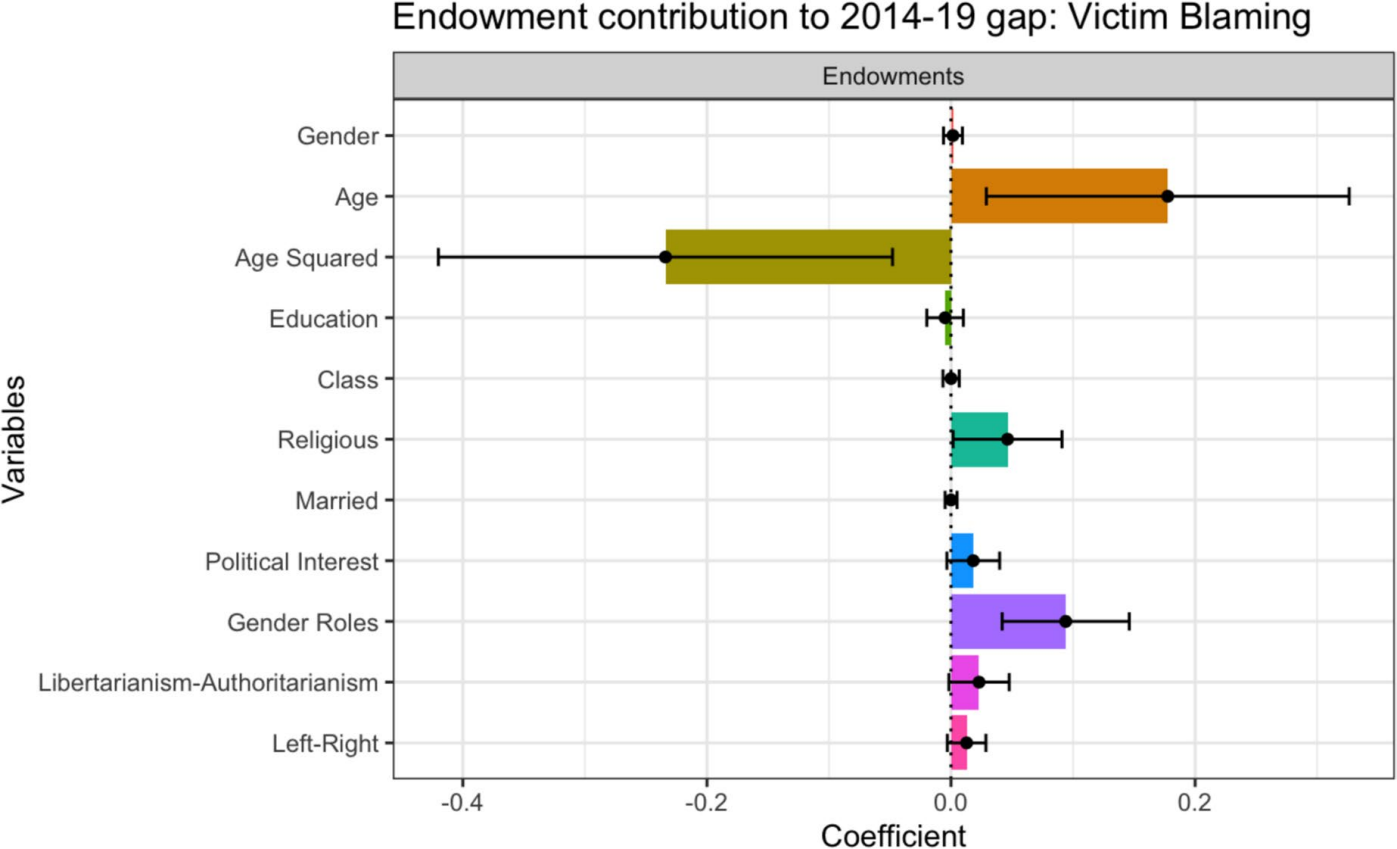
Endowment Effects: Contribution to Gap in Victim Blaming Between 2014 and 2019

As displayed in Table 6, 58.82% of the gap in attitudes is explained by differences in characteristics between the 2014 and 2019 samples, indicating that the 2019 sample would blame the victim more if they had the same characteristics as 2014. The effect of the samples’ differing attitudinal/behavioral characteristics composes 64.63%, and contribute positively to the gap (see Fig. 3). In Fig. 3, a positive coefficient suggests that the level of victim blaming amongst the 2019 sample would increase if it had the same endowments as the 2014 sample. Therefore, if the 2019 sample had the same level of Political Interest, Gender Role Attitudes, Libertarianism-Authoritarianism, and Left–Right Political Orientation as the 2014 sample, they would victim blame more. These findings offer support for H2. Among these effects, Gender Role Attitudes were significant (0.09***), reflecting how the 2019 sample’s less traditional Gender Role Attitudes facilitate lower levels of Victim Blaming. Demographic variables contribute minimally (−5.81%), with significant effects observed for age, age squared, and religious identity (*p* < .05). As shown in Fig. 3, age and age squared have opposite effects, suggesting a small net impact. Meanwhile, the effect of being religious was positive, suggesting that the higher proportion of non-religious individuals in 2019 contributes to the sample’s decreased Victim Blaming.

#### Coefficient Effects

**Fig. 4:**
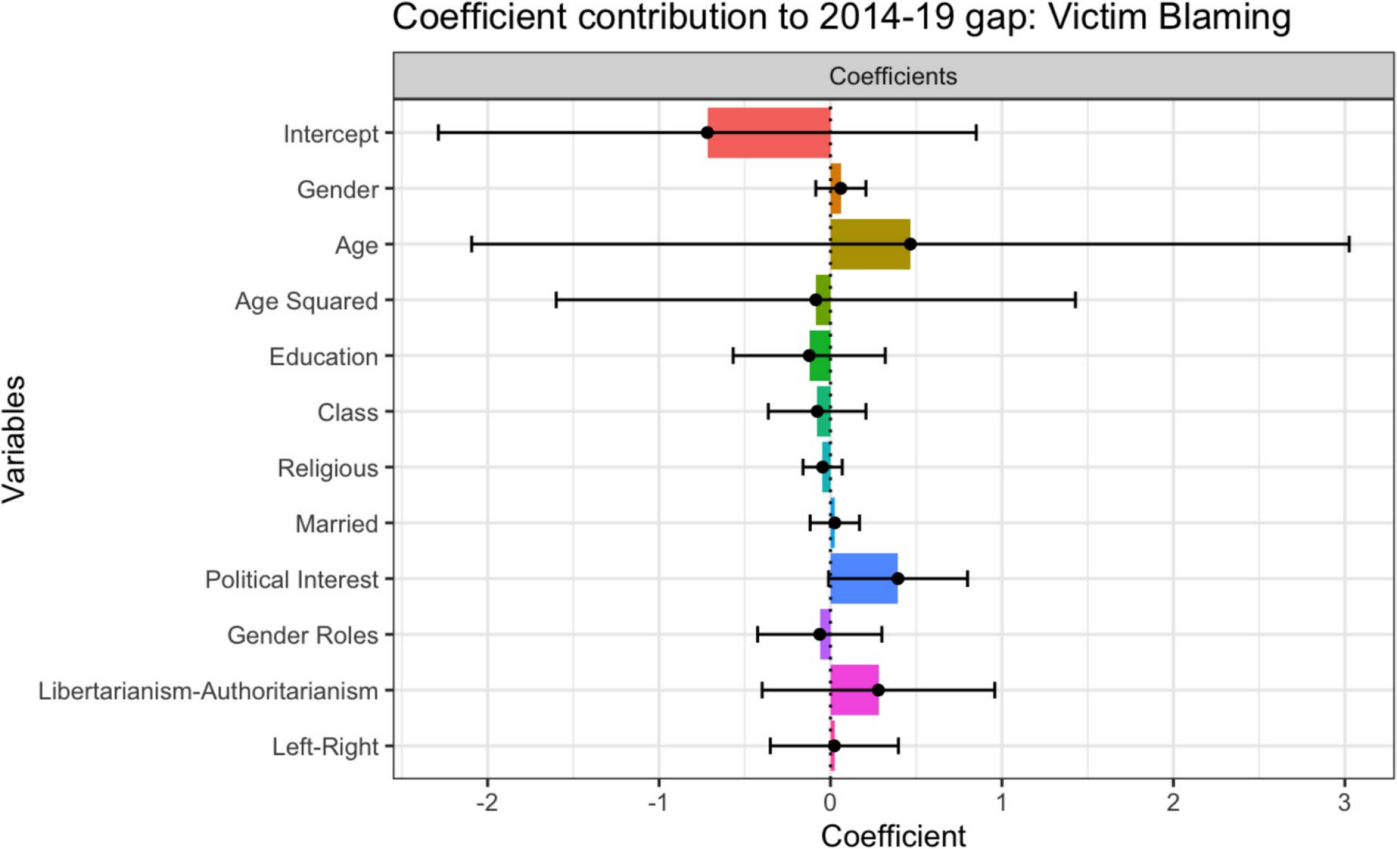
Coefficient Effects: Contribution to Gap in Victim Blaming Between 2014 and 2019

The differing effects of the sample characteristics in 2014 and 2019 account for 58.34% of the difference in Victim Blaming scores. Therefore, if the 2019 sample’s characteristics had the same effects as in 2014, they would victim blame more. These results support H3a. However, no individual variable was significant. Attitudinal and behavioral variables positively contributed to the gap, implying that people in 2019 would victim blame more if their attitudes and political interest had the same effects as in 2014. There was a significant relationship between Political Interest and lower levels of Victim Blaming in 2019, but this variable had a minimal effect in 2014. As shown in Fig. 4, the coefficient effect for Political Interest had the largest effect out of all the attitudinal and behavioral variables and was the closest to being statistically significant (*p* = .057). This result offers limited support for H3bii.

#### Endowment x Coefficient Interaction Effects

**Fig. 5:**
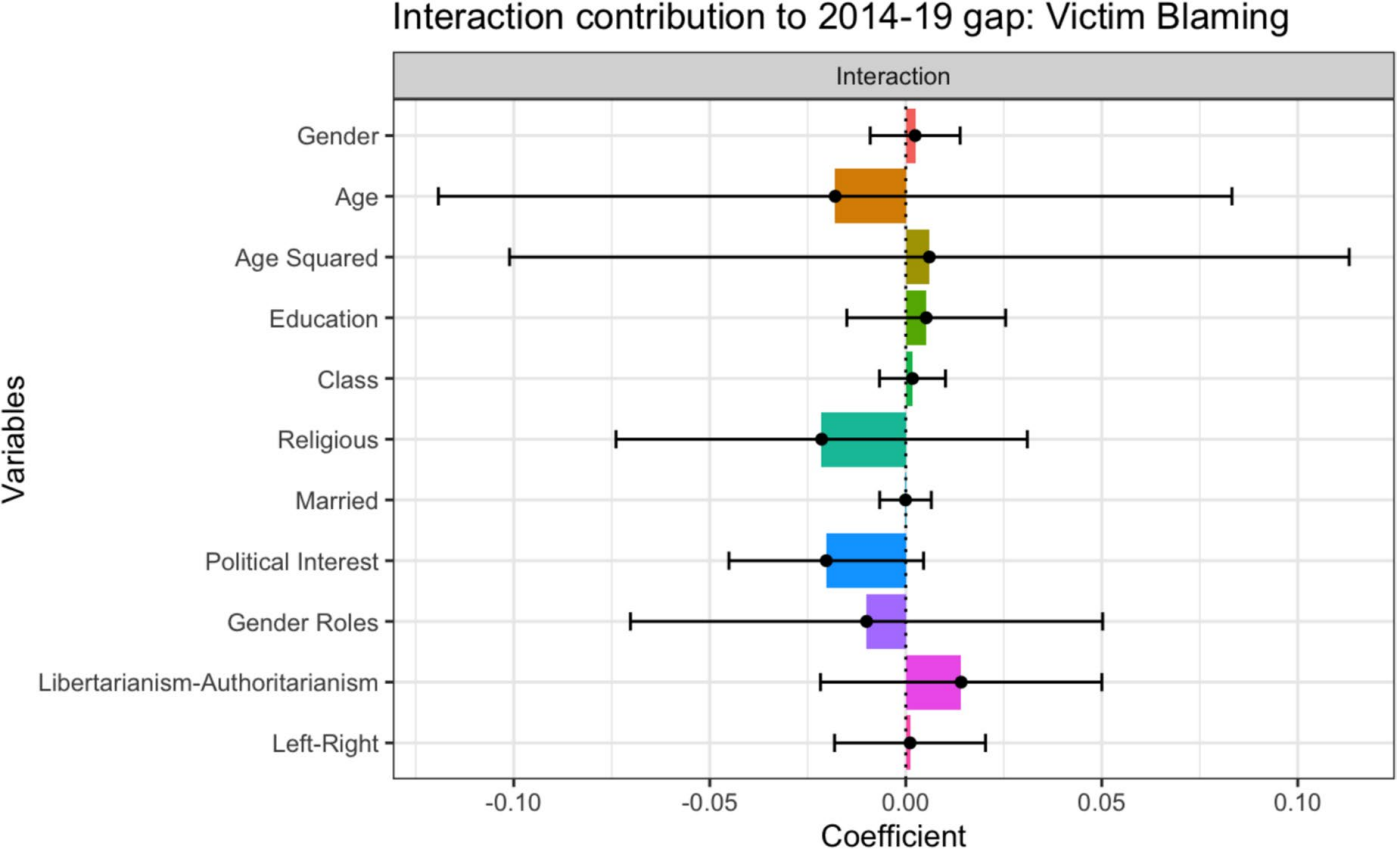
Endowment x Coefficient Interaction Effects: Contribution to Gap in Victim Blaming Between 2014 and 2019

The interaction effect was non-significant, and no variable had a significant effect. As shown in Fig. 5, religious identity, Political Interest, Gender Role Attitudes and age all contribute negatively to the gap. These findings suggest that if the 2019 sample’s endowments and coefficients simultaneously changed to those of the 2014 sample, they would blame the victim less, consistent with H2 and H3a.

#### Summary

Overall, the more liberal social attitudes amongst the 2019 sample – particularly Gender Roles Attitudes – are key in accounting for their decreased Victim Blaming. These findings offer support for H2. Additionally, the larger, negative effect of Political Interest in 2019 contributes to this decline in Victim Blaming, offering support for H3bii. Ultimately, these findings indicate that the decreased Victim Blaming in 2019 emerged because of both attitudinal changes, and due to the changing political context.

### Perceived Perpetrator Culpability

The KOB decomposition for perceived Perpetrator Culpability predicted greater perceptions of culpability in 2019, with a mean score of 3.08 in 2014 and 3.54 in 2019, resulting in a gap of −0.46. As displayed in Table 7, the coefficient component was the largest (82.86%) and most significant contributor to the gap. The endowment component accounted for 17.18%, and the interaction component was negligible.

**Table 7:** Summary of Perpetrator Culpability Threefold Decomposition by Variable Group (Brackets Representing Percentage of Gap)

Component/Variable Category	Endowment	Coefficient	Interaction	Total Contribution
Overall Decomposition				
Total contribution	−0.08 (17.18%)	−0.38 (82.81%)	0.001 (−0.20%)	−0.46 (100.00%)
*SE*	0.03	0.04	0.02	
95% CI	(−0.13, −0.03)	(−0.46, −0.30)	(−0.05, 0.05)	
*p*	.003	< .001	.969	
By Variable Group				
Demographic	0.01 (−1.34%)	−0.64 (139.50%)	−0.01 (1.56%)	−0.64 (139.72%)
Attitudinal/Behavioral	−0.09 (18.52%)	0.38 (−82.04%)	0.01 (−1.76%)	0.30 (−65.29%)
Intercept		−0.12 (25.35%)		−0.12 (25.35%)

#### Endowment Effects

**Fig. 6:**
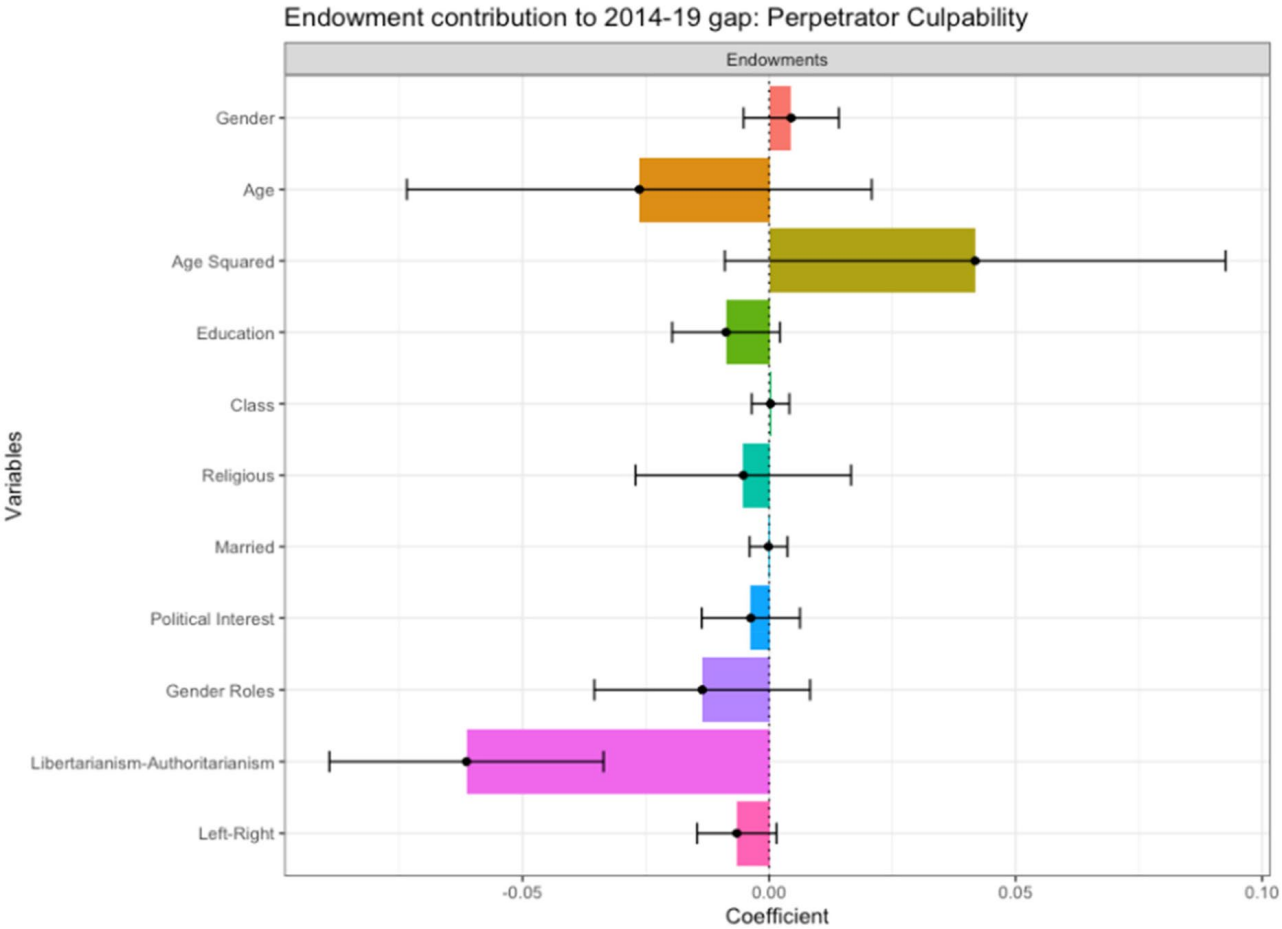
Endowment Effects: Contribution to Gap in Perceived Perpetrator Culpability Between 2014 and 2019

If the 2019 sample had the same characteristics as the 2014 sample, they would have lower perceptions of Perpetrator Culpability, contributing 17.18% to the gap in attitudes (Table 7). This finding supports H2. In the graph below (Fig. 6), a negative coefficient suggests that perceptions of perpetrator culpability amongst the 2019 sample would decrease if it had the same endowments as the 2014 sample. Attitudinal and behavioral variables are the largest contributor to the overall gap (18.52%). Specifically, the 2019 sample’s more libertarian attitudes had a large and significant contribution to their higher perceptions of Perpetrator Culpability (−0.06***), as shown by the large negative coefficient in Fig. 6. Demographic variables contribute minimally to the gap (−1.34%), and none were significant.

#### Coefficient Effects

**Fig. 7:**
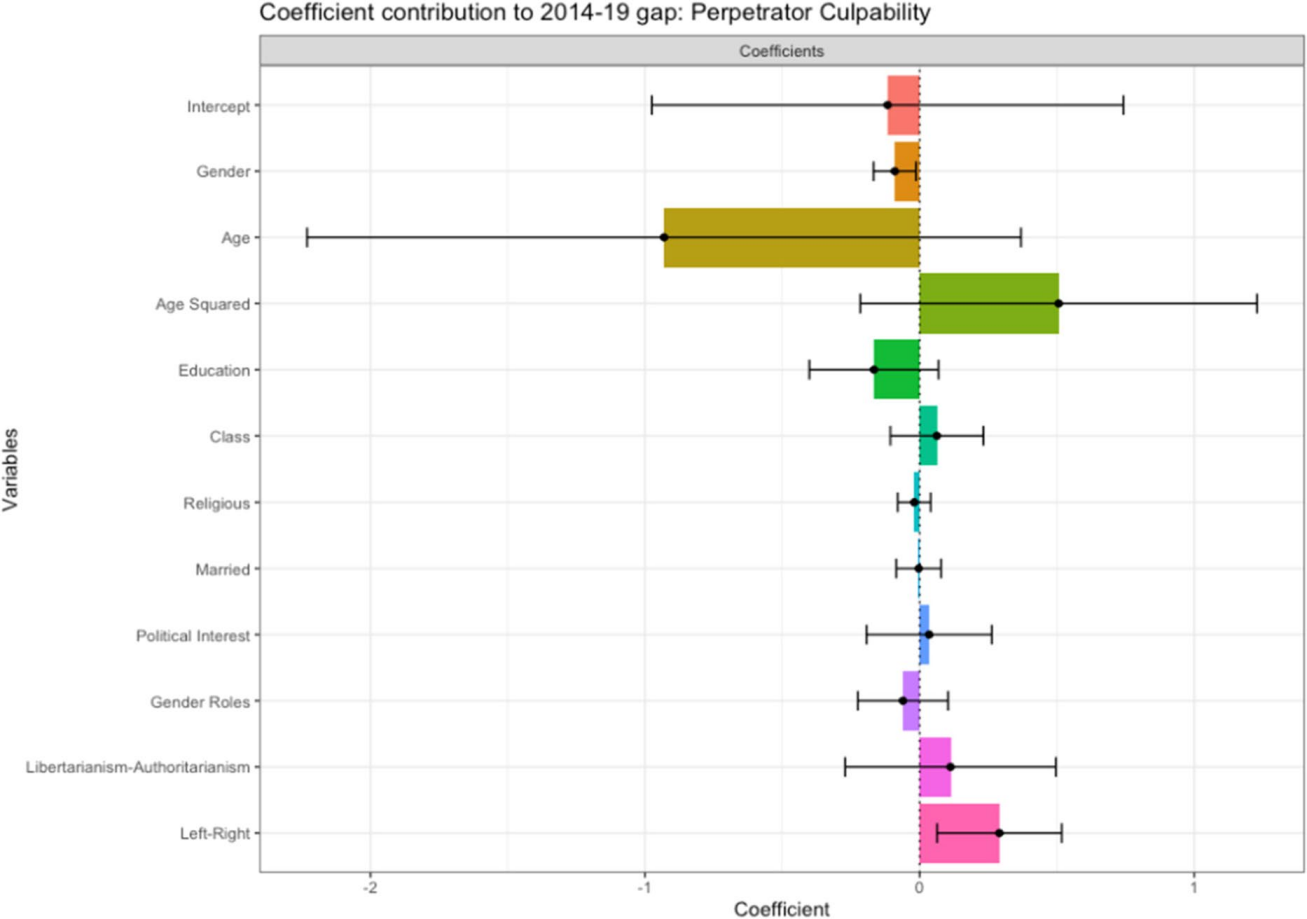
Coefficient Effects: Contribution to Gap in Perceived Perpetrator Culpability Between 2014 and 2019

Differences in the effects of characteristics contribute the most to the gap in attitudes, providing strong support for H3a. Of the coefficient effects, demographic variables are the largest contributors (139.5%), with the effect of being a woman being significant and negative (−0.09*). If being a woman had the same effect on attitudes as it did in 2014, perceptions of Perpetrator Culpability would be lower (see Fig. 7), supporting H3bi. Of the attitudinal variables, only Left–Right Political Orientation is significant and positive (0.29*). Therefore, the decomposition suggests that the 2019 sample would have higher perceptions of Perpetrator Culpability if Left–Right Political Orientation had the same effect as in 2014. As shown by the previous regression analyses, in 2014, right-wing attitudes increased perceptions of culpability, but this effect was smaller and negative in 2019. These results offer mixed evidence for H3biii, as it may indicate increased consensus about issues of sexual violence across the political spectrum, rather than differing reactions across groups. These complex effects will be further investigated in subsequent moderation analyses.

#### Endowment x Coefficient Interaction Effects

**Fig. 8:**
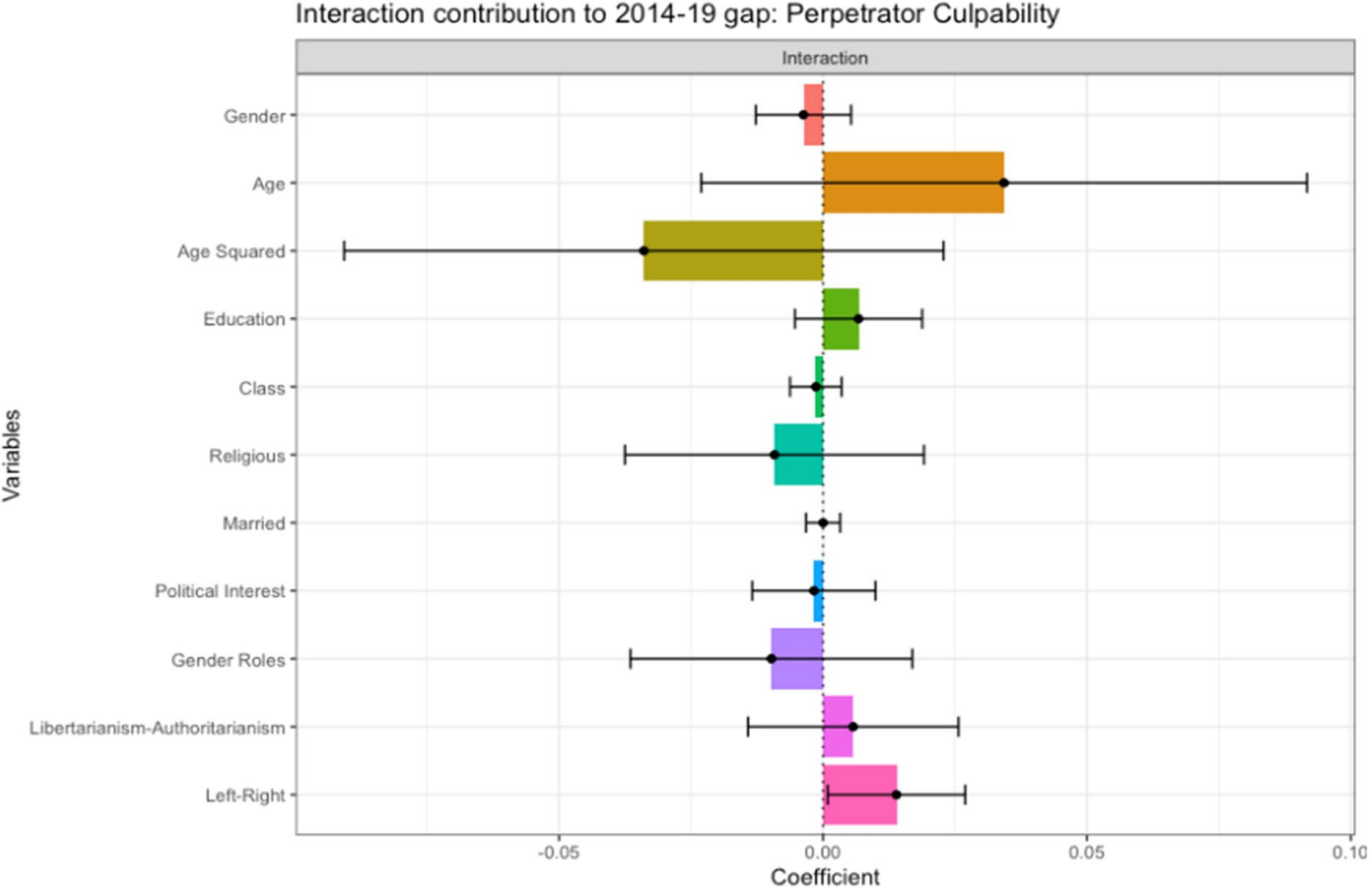
Endowment x Coefficient Interaction Effects: Contribution to Gap in Perceived Perpetrator Culpability Between 2014 and 2019

The interaction between endowments and coefficients had a negligible, non-significant contribution to the gap in attitudes. Attitudinal variables contribute negatively to the gap between 2014 and 2019, while demographic variables contribute positively. The only significant variable is the small positive effect of Left–Right Political Orientation (0.01*), as displayed in Fig. 8. This coefficient indicates that if the 2019 sample were as right-wing as in 2014 (i.e., more right-wing), and if being right-wing had the same (positive) effect on perceived culpability as it did in 2014, the 2019 sample would have slightly higher perceptions of Perpetrator Culpability. These findings will be further investigated in the subsequent moderation analyses.

#### Summary

Overall, the differences in perceptions of Perpetrator Culpability between 2014 and 2019 relate principally to the changing effects of the 2019 sample’s characteristics. Although the more libertarian political attitudes of the 2019 sample contributed positively to their perceptions of culpability (supporting H2), much of the gap in attitudes was driven by the fact that women in 2019 had higher perceptions of Perpetrator Culpability than men, and by the emergence of a negative relationship between right-wing political attitudes and perceived Perpetrator Culpability. These findings offer further evidence for H3bi – women appear to have been influenced by their social context in a way that they were not in 2014. Meanwhile, the significant and varying effect of Left–Right Political Orientation requires further analysis to understand its effects on attitudes, detailed below.

### Moderation Analyses

To further understand the statistically significant coefficient effects observed in the K–O-B decomposition, moderation analysis was conducted to investigate how the relationships between the two dependent variables and independent variables vary across years. Johnson-Neyman intervals were calculated to identify the specific values of the independent variable where the survey year significantly influences its effect on attitudes toward sexual violence. Interaction plots and predicted values were calculated using the ‘interactions’ and ‘emmeans’ R packages (Lenth, 2024; Long, 2024).

### Victim Blaming

#### Political Interest

**Fig. 9:**
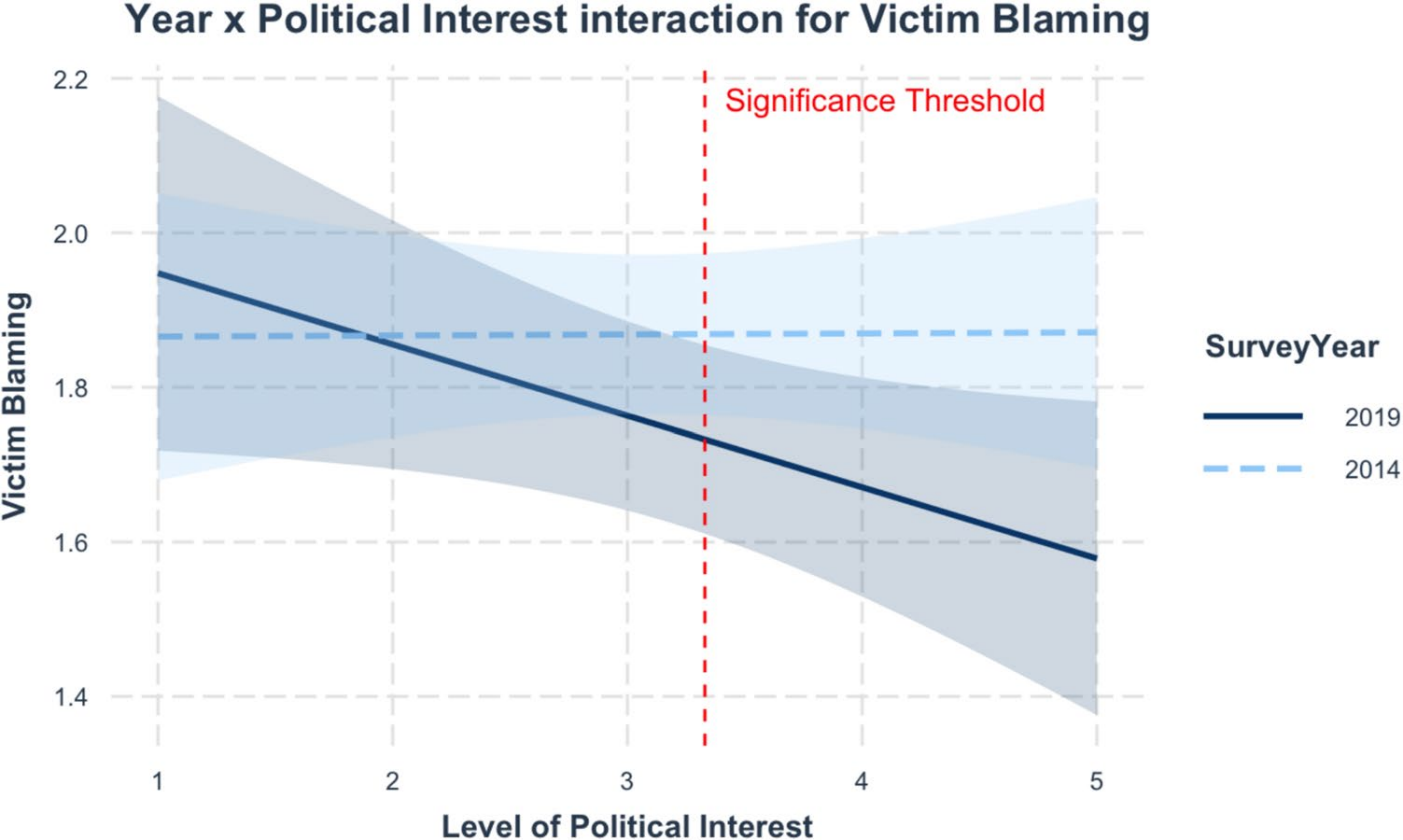
Simple Slopes—Interaction Between Political Interest and Year (Victim Blaming)

The coefficient effect for Political Interest, although not statistically significant (*p* = .057), was investigated due to the large differences in effects between 2014 and 2019, the large coefficient effect observed in the decomposition, and the variability introduced into the estimation of the decomposition’s significance levels by the bootstrapped standard errors. In 2014, Political Interest had a small, positive, and non-significant effect on attitudes (0.01), while in 2019, the effect was negative, large (0.11***), and significant. As shown in Fig. 9, there is only a statistically significant (*p* < .05) effect of the survey year on Victim Blaming when levels of Political Interest are above a score of 3.3, equivalent to more than ‘some interest’ in politics. These finding suggests that the reduction in Victim Blaming in 2019 was influenced by people with high levels of Political Interest being less likely to victim blame than people who were less interested in politics. Indeed, in 2014, Victim Blaming was stable across levels of Political Interest, with just a 0.01 difference between the most and least engaged. By 2019, this gap widened to −0.36. These findings offer support for H3bii – Victim Blaming declined more amongst those with higher levels of political interest.

### Perceived Perpetrator Culpability

#### Left–Right Political Orientation

The effect of Left–Right Political Orientation on perceived Perpetrator Culpability is negative and non-significant in 2019 (−0.06), and positive and significant in 2014 (0.07*). Johnson-Neyman intervals indicate that there are significant (***) differences in perceptions of Perpetrator Culpability between 2014 and 2019 up to a score of 3.54 on the left–right scale. The differences in perceptions of Perpetrator Culpability between 2014 and 2019 were non-significant from a score on the left–right scale of 4.00 upwards. This result indicates that attitudes toward sexual violence changed less at the most right-wing end of the political spectrum. Smaller changes amongst very right-wing individuals may help to explain the non-significant effect of Left–Right Political Orientation in 2019 – if left-wingers’ attitudes changed more than right-wingers’, then this would have the effect of reducing differences in perceptions of perpetrator culpability across the political spectrum.

**Fig. 10:**
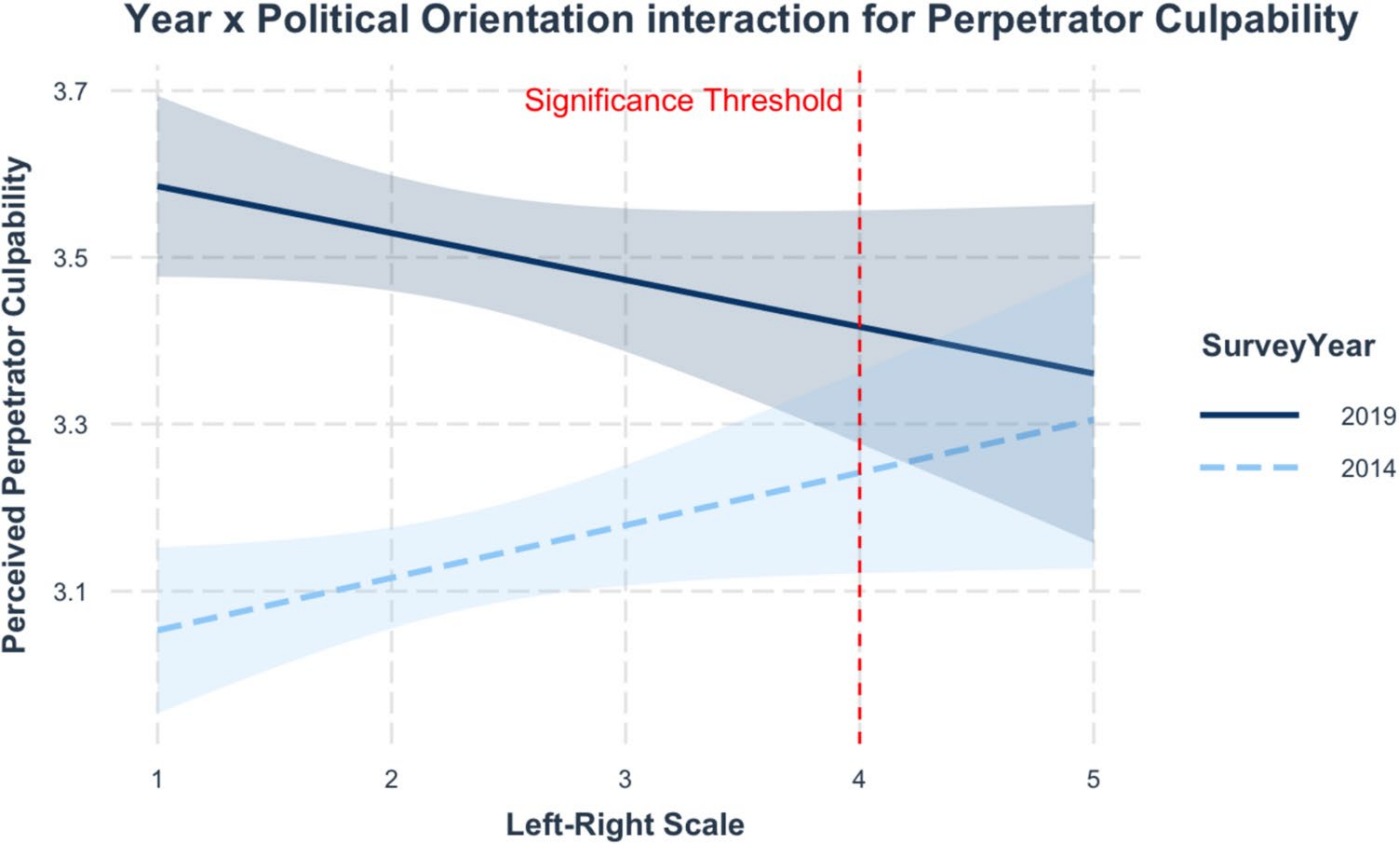
Simple Slopes—Interaction Between Left–Right Political Orientation and Year (Perpetrator Culpability)

Indeed, Fig. 10 shows that perceptions of Perpetrator Culpability vary greatly between 2014 and 2019 at the left-wing end of the scale, whereas the gap is very small at the right-wing end of the scale. Calculation of predicted values suggests that this amounts to a significant 0.54 difference between 2014 and 2019 at the most left-wing end of the scale. In contrast, there was only a non-significant difference of 0.05 between 2014 and 2019 at the most right-wing end of the scale. These findings suggest that left-wingers’ attitudes were more responsive to the changing political environment than right-wingers, offering support for H3biii.

#### Gender

**Fig. 11:**
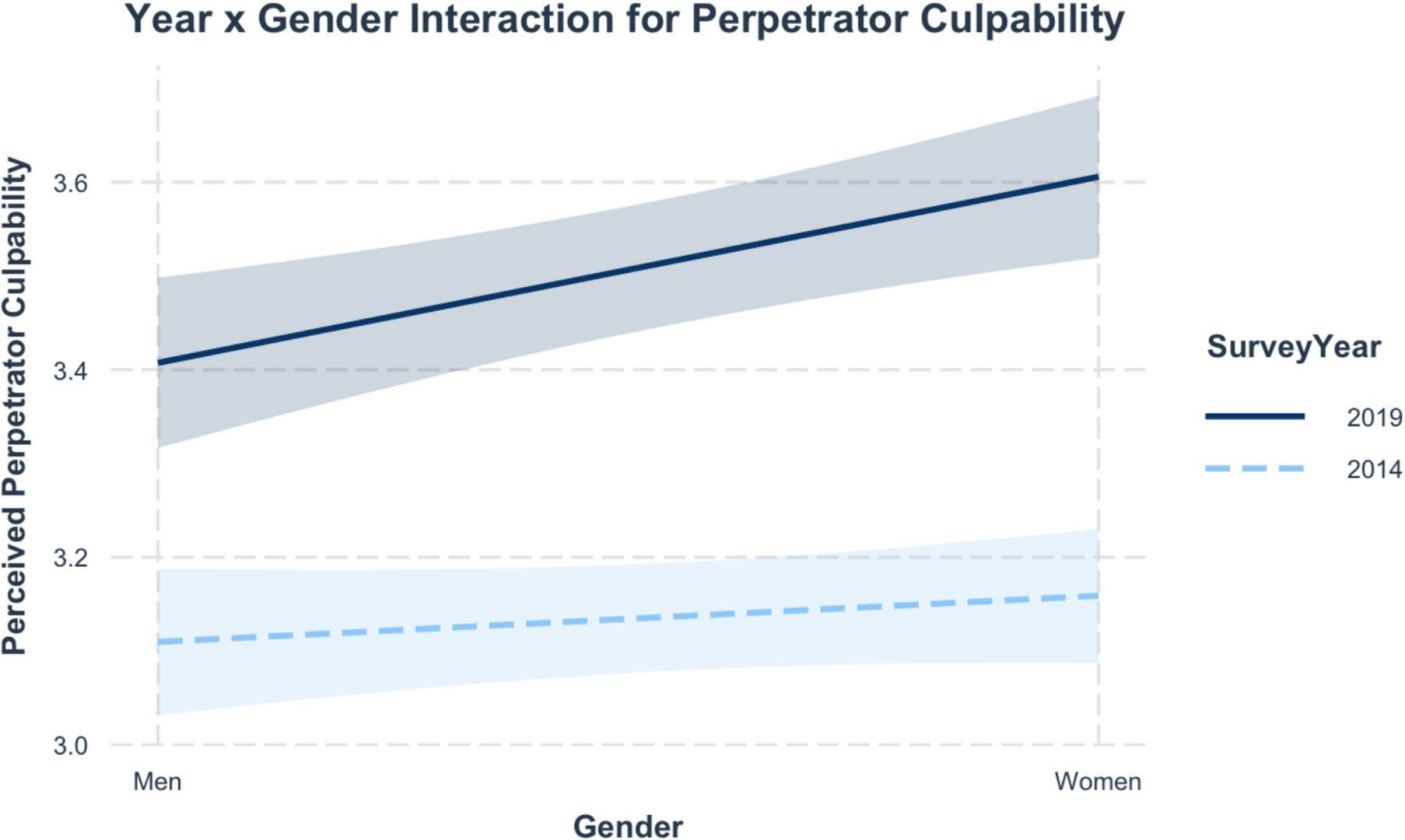
Simple Slopes—Interaction Between Gender and Year (Perpetrator Culpability)

The KOB decomposition reveals a significant coefficient effect (−0.09*) for gender – being a woman had a larger, significant effect on perceived Perpetrator Culpability in 2019 (0.20***), and a small, non-significant effect in 2014 (0.035). Johnson-Neyman intervals calculated to estimate the significance of the effect of survey year on men and women’s attitudes indicate that there were significant changes in perceptions of Perpetrator Culpability for both men (slope = 0.30, *p* < .001) and women (slope = 0.45, *p* < .001). However, there were significant differences between men and women’s scores in 2019, as indicated by the significant slope of gender in 2019 (0.2, *p* < .001). These patterns are displayed in Fig. 11, which shows a larger gender gap in attitudes in 2019. Predicted values suggest that men’s perceptions of Perpetrator Culpability only increased by 0.29, compared to a 0.54 increase in women’s scores. Therefore, while both men and women’s attitudes changed significantly between 2014–19, in 2019 a significant gender gap emerged in which women had higher perceptions of Perpetrator Culpability than men. These results supports H3bi, suggesting a larger increase in perceptions of Perpetrator Culpability for women, who generally have greater identification with and exposure to issues of sexual violence. No significant differences by gender were observed for Victim Blaming, indicating that group-based differences in attitudes vary across dimensions of rape myths.

A summary of the hypotheses tested in this study and whether they were supported is provided in Table 8 below.

**Table 8:** Summary of Hypothesis Testing Results

Hypothesis	Supported?	Evidence
H1: Victim blaming decreased, and perceptions of perpetrators’ culpability increased between 2014 and 2019	Yes	T-tests show significant differences in means between 2014–19 – the 2019 sample victim blamed less and perceived greater perpetrator culpability
H2: The liberalization of social attitudes — specifically political orientation and gender role attitudes — accounts for a significant proportion of the change in attitudes towards sexual violence between the 2014 and 2019 samples	Yes	Decomposition shows significant endowment effect for gender role attitudes (victim blaming) and liberalism-authoritarianism orientation (perpetrator culpability)
H3a: A significant proportion of the change in attitudes towards sexual violence is explained by the differing effects of predictor variables in 2014 compared to 2019	Yes	Decomposition shows significant coefficient effect for both victim blaming and perceived perpetrator culpability
H3b: Predictors that may reflect exposure to and identification with #MeToo will have stronger effects in 2019 than in 2014. This will explain a proportion of the change in attitudes towards sexual violence. These include: i) being a woman, ii) political interest, iii) left-wing/libertarian political orientation	Partially	Decomposition shows significant coefficient effects for being a woman (H3bi) and left–right orientation (H3biii) on perceptions of perpetrator culpability. Moderation analyses suggest significant variation in the effect of being more politically engaged (H3bii), left-wing and a woman between 2014–19

## Discussion

This study explored changes in attitudes towards sexual violence in Scotland between 2014 and 2019 to examine whether differences in attitudes were attributable to changes in sample characteristics, or to varying effects of their characteristics across years. The study tested the hypotheses that changes in attitudes toward sexual violence were explained by both the liberalization of social attitudes, and by the changing effects of characteristics inferred to be associated with greater interaction with #MeToo.

The findings, summarized in Table 8, largely support the hypotheses. In line with H1, there were significant changes in attitudes toward sexual violence between 2014 and 2019. Levels of victim blaming declined by 3.44% and perceptions of perpetrator culpability increased by 9.06%. In support of H2, the 2019 sample’s more libertarian political and gender-role attitudes contributed to changes in attitudes. In support for H3a and partial support for H3b, the changing effects of key predictors explained a significant proportion of the gap in attitudes. Moreover, the decomposition analysis revealed unexpected but theoretically consistent evidence of the role of differing factors in the changes in attitudes towards victim blaming and perpetrator culpability. These results were largely consistent with existing literature highlighting how changes in attitudes toward sexual violence are shaped by a) liberalization of societal attitudes, and b) group-based exposure to and mobilization by the #MeToo movement.

### Victim Blaming

Shifts in attitudes predictive of beliefs about sexual violence were a central driver of changes in victim blaming between 2014–19. There was a significant shift in gender role attitudes between 2014 and 2019, with the 2019 sample having less traditional gender role attitudes. This is consistent with long-term trends of increasing egalitarianism in the United Kingdom (Park et al., 2013). The KOB decomposition demonstrates that the 2019 sample’s less traditional gender role attitudes contributed significantly to their decreased victim blaming. This endowment effect provides evidence for H2 and supports existing literature highlighting the relationship between victim blaming and traditional gender roles (Agadullina et al., 2022; Chapleau et al., 2007; Gothreau et al., 2022; Koepke et al., 2014; Masser et al., 2010; Yamawaki, 2007). The items used in the present study to measure victim blaming evaluate assumptions about acceptable gendered behavior, including whether a woman is to blame for rape if she drinks alcohol or wears revealing clothes. In 2019, declining gender traditionalism may have challenged assumptions about ‘appropriate’ behavior for women. This process of attitudinal change could have undermined perceptions that drinking alcohol and wearing revealing clothes are deviant behaviors that legitimize sexual violence. Thus, the findings are consistent with the argument that liberalization of social attitudes contributed to shifts in perceptions of sexual violence.

The large, significant contribution of coefficient effects to changes in victim blaming suggests that a proportion of the gap in attitudes emerges from the diverging effects of key characteristics across the samples. The decomposition and moderation analyses suggest that differing levels of victim blaming are partially explained by the larger and more significant effect of having high levels of political interest on victim blaming in 2019. These results supports H3bii. The changing effects of political interest on victim blaming may reflect the increased debate about sexual violence after the #MeToo movement. Politically engaged individuals would have been more exposed to these debates after 2014, challenging traditional sexual scripts that influence interpretations of rape. This theorized mechanism aligns with evidence that exposure to the #MeToo movement produced greater opposition to sexual violence (Armstrong & Mahone, 2023; Palmer et al., 2021; Szekeres et al., 2020).

### Perceptions of Perpetrator Culpability

Perceptions of perpetrator culpability were influenced by a combination of sample characteristics, and the changing effects of those characteristics between 2014 and 2019. The decomposition reveals a significant association between the more libertarian political attitudes of the 2019 sample and their heightened perceptions of culpability, offering evidence for H2. The role of the 2019 sample’s greater libertarianism in explaining their greater perceptions of perpetrator culpability reflects evidence from previous studies highlighting the relationship between system justification, authoritarianism and attitudes that deny rape allegations and emphasize male perpetrators’ innocence (Bareket & Fiske, 2023; Martini & De Piccoli, 2020). The 2019 sample’s greater libertarianism may have facilitated less attachment to existing hierarchies and systems, therefore increasing perceptions of perpetrator culpability. This finding is consistent with the theoretical expectation that the liberalization of social attitudes may challenge rape myth acceptance.

However, most of the gap between the 2014 and 2019 samples’ perceptions of perpetrators’ culpability was explained by the differing effects of key predictors between survey years, supporting H3a. Notably, the large, positive effect of being a woman in 2019 significantly contributed to the gap in attitudes. As shown by the moderation analysis, women experienced a larger increase in perceived perpetrator culpability compared to men, supporting H3bi. Similarly, the differing effect of left–right political orientation between 2014 and 2019 contributed to the differences in the two groups’ attitudes. The moderation analysis reveals a large increase in perceived perpetrator culpability at the left-wing end of the scale in 2019 compared with minimal differences between years on the right, offering some support for H3biii.

These findings are consistent with the theoretical expectation of diverging responses to issues of sexual violence following the #MeToo movement and may indicate differences in engagement with the movement. The changing effects of political orientation and gender reflect evidence that there was greater participation in and support for the #MeToo movement amongst women and left-wingers (Armstrong & Mahone, 2023; Hansen & Dolan, 2023; Herrera Hernandez & Oswald, 2022; Hoffman, 2021; Kunst et al., 2018; Lisnek et al., 2022; Szekeres et al., 2020).

Beyond differing levels of *exposure* to #MeToo, the results of the decomposition and moderation analyses could also indicate differing *reactions* to the movement. The smaller changes in men and right-wing people’s attitudes relative to women and left-wingers could indicate some resistance to the changes precipitated by #MeToo. The items used to measure perceptions of perpetrator culpability invoked contentious issues – the statement that *“women often lie about being raped”* presents them as malicious and deceitful, while the statement that *“rape results from men being unable to control their need for sex”* portrays men are innocent and helpless. The issues of male responsibility and false accusations are associated with hostile sexism, which antagonizes women while emphasizing men’s righteousness. These psychological processes are shown to facilitate attitudes that legitimize sexual violence (Abrams et al., 2003; Bohner et al., 2009; Rollero & Tartaglia, 2018).

Such attitudes became highly salient within public discourse after the #MeToo movement. Hashtags like #BelieveWomen emphasized the rarity of false rape accusations, and trends like #HowIWillChange placed increased responsibility on men to change their behavior (PettyJohn et al., 2019; Serisier, 2022). In 2014 (prior to #MeToo), these issues received less attention. Therefore, questions measuring perceptions of culpability relate to political debates that were louder and more contentious in 2019. Studies of the #MeToo movement’s impact on attitudes toward sexual violence suggests increased gender divisions and hostility toward women due to anxiety over sexual assault allegations (Hansen & Dolan, 2023; Kunst et al., 2018; Lisnek et al., 2022; Nutbeam & Mereish, 2022). Therefore, issues of perpetrator culpability may have induced greater status anxiety and feelings of collective responsibility amongst some men, discouraging attitude change.

Meanwhile, the stability in perceptions of perpetrator culpability at the most right-wing end of the political orientation scale may reflect the association between right-wing attitudes and system-justifying beliefs that reject the need for social change (Duckitt & Sibley, 2009; Jedinger & Kaminski, 2024; Jost & Hunyady, 2005). In the context of rapid social shifts triggered by the #MeToo movement, right-wingers’ system justification may have provided motivation to protect existing norms and hierarchies, reducing their likelihood of attitude change. Ultimately, although these results align with theoretical expectations of political and social groups’ diverging responses to #MeToo, they highlight the need for further research into backlash against the movement and its impact on attitudes toward sexual violence.

### Limitations and Future Research Directions

Despite its strengths, this work has limitations. There is a dearth of longitudinal studies on attitudes towards sexual violence, both in the United Kingdom and globally, and the few options available either lack detail or accessibility (American National Election Studies, 2021, 2025; Coumarelos et al., 2023). Whilst limited by use of repeated-cross-sectional surveys rather than panel data, this study has the advantage of providing both sufficient statistical power and generalizability to a whole nation’s population. This methodology improves upon that of most studies of attitudes toward sexual violence, which employ convenience samples of college students (Beshers & DiVita, 2021; Byrne et al., 2021). The surveys’ cross-sectional design means that the evidence speaks to the plausibility of possible causes of attitude change but cannot be definitive. For example, changes in attitudes may reflect short-term variation, but not longer-term trends towards more progressive attitudes. The lack of specific indicators for exposure to #MeToo also limits the ability to establish causal links between attitudinal shifts and these specific factors. Nonetheless, the Kitagawa-Oaxaca-Blinder decomposition analysis goes some way toward testing the theoretical proposition that changes in the political environment influenced people’s attitudes toward sexual violence between 2014–19. Future research would benefit from larger-scale collection of panel data on attitudes towards sexual violence, with more investigation into the discursive and informational sources from which individuals derive their understandings of the issue.

### Practice Implications

The present study offers several insights for efforts to combat sexual violence. Firstly, by generating insights into which groups’ attitudes have been most impacted by social change, the study illuminates which sectors of a population may benefit from efforts to improve their understanding of sexual violence. Additional strategies may be required to respond to the backlash against social movements like #MeToo. Consent educators and policymakers should consider effective ways to communicate information about sexual violence and rape myths to groups who may be more prone to endorsing these attitudes. A process of targeted intervention is particularly relevant given the evidence of a shift toward the far-right and support for misogynistic influencers amongst young men and boys (Burn-Murdock, 2024; Campbell et al., 2024; Cox, 2023; Haslop et al., 2024; Horeck et al., 2023).

Society-wide decline in rape myth acceptance is important for reducing the perpetration of sexual violence and ensuring an environment that is conducive to survivors’ recovery. Rape myth acceptance is associated with greater perpetration of sexual violence, lower likelihood of reporting sexual crimes, and inadequate institutional responses (Heath et al., 2013; Holland et al., 2020; Trottier et al., 2019; Venema, 2019; Yapp & Quayle, 2018). Therefore, the decline in rape myth acceptance may result in people becoming more likely to practice consent, call out problematic behavior, or offer appropriate support to those who have been victimized. The present study indicates a need for interventions to ensure that broad changes in social attitudes are manifested in individuals’ sexual and interpersonal behavior. Practitioners who offer interventions such as consent training and active bystander training may wish to demonstrate how rape myths may manifest in interpersonal behavior and offer strategies to challenge such attitudes within sexual interactions.

Finally, the study highlights that there can be different demographic, attitudinal, and contextual drivers of attitudes toward victim blaming that are distinct from those influencing perceptions of perpetrator culpability. These findings underline the need for consent education to address multiple dimensions of attitudes toward sexual violence.

## Conclusion

This study examined shifts in attitudes toward sexual violence in Scotland between 2014 and 2019, revealing significant changes in how rape is understood. Over time, people became less likely to blame victims and more likely to hold perpetrators responsible. These patterns are consistent with theoretical expectations that attitudinal change stems from both the liberalization of social values and groups’ varying engagement with new public discourses on sexual violence, such as those shaped by the #MeToo movement. The decrease in support for traditional gender roles, coupled with the increasing significance of political interest as a predictor of reduced victim blaming, may indicate shifts away from narratives about sexual violence rooted in traditional gender norms, toward alternative frameworks promoted within contemporary political discourses. Meanwhile, changes in how gender and political ideology influence perceptions of perpetrator culpability point to group-specific responses to the increased discussion of sexual violence in 2019 compared with 2014.

As the first study to use the Scottish Social Attitudes Survey to explore beliefs about sexual violence, this research provides important theoretical and empirical contributions. It demonstrates that attitudes toward sexual violence are shaped by evolving social norms and political discourse, which have uneven effects across society. Beyond Scotland, further research is required to examine whether there have been similar changes in perceptions of sexual violence within other contexts, particularly those with more rigid gender roles or weaker feminist activism. Ultimately, these findings expose significant societal shifts in perceptions of sexual violence, as well as the fragmented nature of this social change.

## Supplementary Information

Below is the link to the electronic supplementary material.

Supplementary file1 (DOCX 432 KB)

## Data Availability

The SSAS data files are accessible via the UK Data Service (ScotCen Social Research, 2016, 2023).
