# Supplementary material for: Explaining Societal Shifts in Victim Blaming and Perpetrator Culpability for Sexual Violence: Evidence From the #MeToo Era
Source: Sex Roles. 2025 Jul 7;91(7):50. doi: 10.1007/s11199-025-01590-6 (PMC12234629; doi:10.1007/s11199-025-01590-6)
Supplement: Supplementary file 1 — Supplementary file1 (DOCX 432 KB) [file 11199_2025_1590_MOESM1_ESM.docx]

# **Supplementary Materials**

Table of Contents

[Supplementary Materials 1](#_Toc196062070)

[1. Google Trends Graphs 2](#_Toc196062071)

[2. Variables 4](#_Toc196062072)

[2.1 Dependent Variables 4](#_Toc196062073)

[2.2 Attitudinal Covariates: 4](#_Toc196062074)

[2.3 Demographic Variables: 6](#_Toc196062075)

[3. Kitagawa-Oaxaca-Blinder Decomposition 7](#_Toc196062076)

[3.1 K-O-B Decomposition: Victim Blaming 7](#_Toc196062077)

[3.2 K-O-B Decomposition: Perpetrator Culpability 10](#_Toc196062078)

[4. Models used for moderation analysis 13](#_Toc196062079)

[4.1 Victim Blaming 13](#_Toc196062080)

[4.2 Perpetrator Culpability 15](#_Toc196062081)

[References 16](#_Toc196062082)

## **Google Trends Graphs**


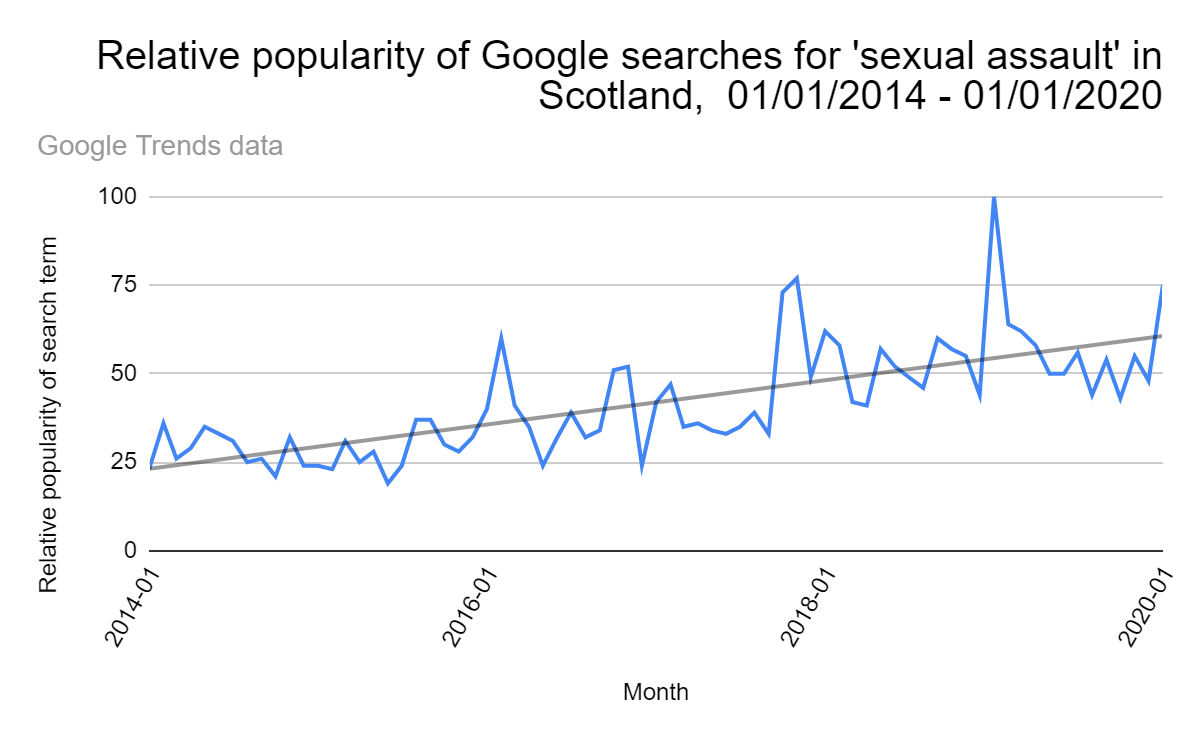


*Figure A: Searches for 'Sexual Assault' in Scotland, 2014-2020 (Google Trends data)*


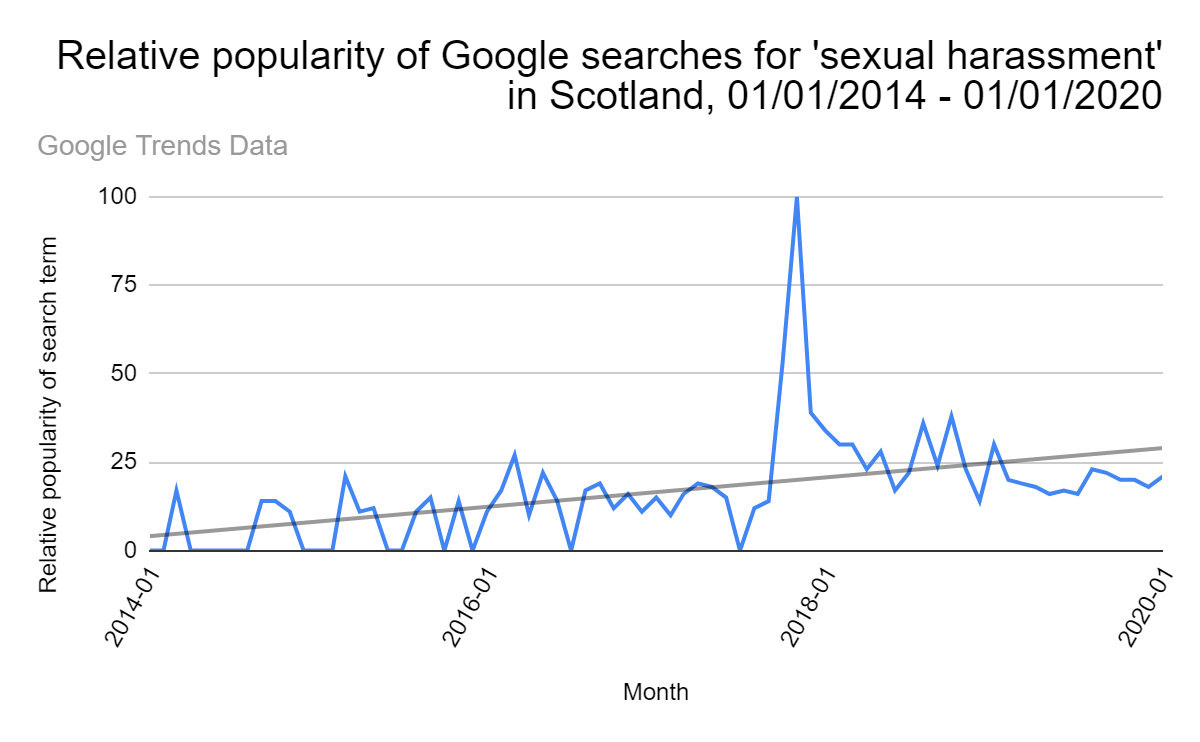


*Figure B: Searches for 'Sexual Harassment in Scotland, 2014-2020 (Google Trends data)*


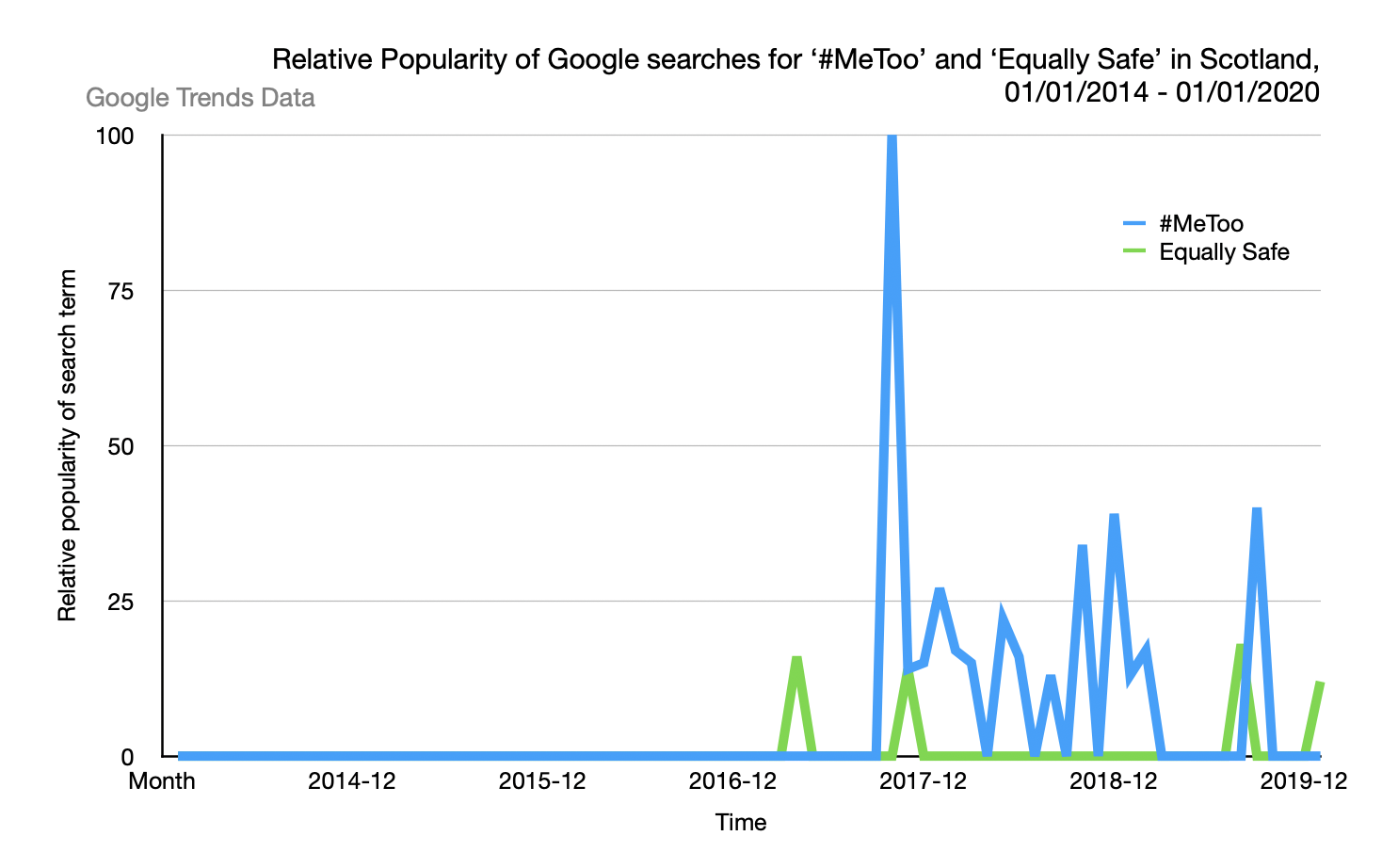


*Figure C: Searches for ‘#MeToo and ‘Equally Safe’ in Scotland, 2014-2020 (Google Trends data)*

This data from Google Trends shows that #MeToo received 15.5 times as much interest as Equally Safe on Google. Since the launch of Equally Safe in June 2014, its average level of interest was 0.88, compared with an average of 13.4 for #MeToo since its launch in October 2017.

## **2. Data and Variables**

The total sample size from the merged datasets was 2,523 people, comprising 1,498 from the 2014 sample and 1,022 from the 2019 sample. Because the 2019 survey included 16-17-year-olds, these individuals were excluded to match the 2014 sample. Individuals with missing data for the dependent variables and covariates were also excluded. Survey weights were not used because the 2014 and 2019 datasets did not have weights with equivalent functions. There is an absence of information about how the weights are calculated in the surveys’ technical reports. Descriptive statistics (displayed in Table 1) show that the 2014 and 2019 samples have broadly comparable demographic characteristics.

### **2.1 Dependent Variables**

#### Confirmatory factor analysis

| Model |  | Df | *p*^^[[1]](#footnote-1)^^ | CFI | TLI | RMSEA |
| --- | --- | --- | --- | --- | --- | --- |
| Main two-factor model (Victim Blaming and Perpetrator Culpability) | 1.143 | 6 | 0.285 | 1.000 | 1.000 | 0.008 |

#### Multiple-group confirmatory factor analysis

| Model |  | Df | *p* | CFI | TLI | RMSEA |
| --- | --- | --- | --- | --- | --- | --- |
| Configural invariance | 1.533 | 10 | 0.821 | 1.000 | 1.008 | 0.000 |
| Metric invariance | 8.293 | 10 | 0.600 | 1.000 | 1.002 | 0.000 |
| Scalar invariance | 21.976 | 16 | 0.144 | 0.997 | 0.995 | 0.026 |
| Strict invariance | 47.694 | 28 | 0.012 | 0.989 | 0.991 | 0.035 |

### **2.2 Attitudinal Covariates:**

#### Political Interest:

This variable was labelled ‘Politics’ in the survey documentation. The variable was then reverse scored, so that a higher score represented higher levels of political interest.

How much interest do you generally have in what is going on in politics?

1. A great deal
2. Quite a lot
3. Some
4. Not very much
5. None at all

#### Gender Role Attitudes:

The ‘Gender Role Attitudes’ variable was an average of two variables found in the 2014 and 2019 SSAS, labelled ‘GenRolB’ and ‘GenRolG’.

GenRolB:

- 1. Imagine you are taking a 3 year old boy to a shop to buy a toy. When you get there he picks a princess doll. Which of the phrases below best describes what you would do?
     - 1. Buy it for him without saying anything
       2. Buy it, but first try to get him to pick a toy that’s more common for boys
       3. Make him put the doll back and pick a toy more common for boys

GenRolG:

- 1. Imagine you are taking a 3 year old girl to a shop to buy a toy. When you get there she picks a toy truck. Which of the phrases below best describes what you would do?
     - 1. Buy it for her without saying anything
       2. Buy it, but first try to get her to pick a toy that’s more common for girls
       3. Make her put the truck back and pick a toy more common for girls

#### Libertarianism-Authoritarianism Political Orientation

Libertarian-Authoritarian Political Orientation was measured using a scale derived from six different items within the SSAS, using the variable ‘libauth’.

1. 'Young people today don't have enough respect for traditional British values'
2. 'People who break the law should be given stiffer sentences'
3. 'For some crimes, the death penalty is the most appropriate sentence'
4. ‘Schools should teach children to obey authority'
5. 'The law should always be obeyed, even if a particular law is wrong'
6. ‘Censorship of films and magazines is necessary to uphold moral standards'

#### Left-Right Political Orientation

Left-Right Political Orientation was measured using a scale derived from five different items within the SSAS, using the variable ‘leftrigh’.

1. ‘Government should redistribute income from the better-off to those who are less well off'
2. ‘Big business benefits owners at the expense of workers'
3. 'Ordinary working people do not get their fair share of the nation's wealth'
4. 'There is one law for the rich and one for the poor'
5. 'Management will always try to get the better of employees if it gets the chance'

### **2.3 Demographic Variables:**

The decomposition breaks down the effect of each variable into the endowment, coefficient and interaction effect, which makes it somewhat sensitive to small sample sizes. Scale variables were used for class and education to preserve statistical power, and curve estimation, correlations, and linear and logistic regression confirmed that treating class and education as scalar variables yields equivalent results for the two dependent variables as when the covariates are treated as categorical.

# **3. Kitagawa-Oaxaca-Blinder Decomposition**

**3.1 K-O-B Decomposition: Victim Blaming**

*Endowment component of decomposition for Victim Blaming*

| Variable | Coefficient | *SE* | 95% CI | *p* |
| --- | --- | --- | --- | --- |
| Gender | 0.002 | 0.004 | (-0.006, 0.009) | 0.691 |
| Age | 0.178 | 0.076 | (0.029, 0.326) | 0.019 |
| Age Squared | -0.234 | 0.095 | (-0.420, -0.048) | 0.014 |
| Education | -0.005 | 0.008 | (-0.020, 0.010) | 0.524 |
| Occupational Class | <0.001 | 0.003 | (-0.007, 0.007) | 0.986 |
| Religious | 0.046 | 0.023 | (0.002, 0.091) | 0.042 |
| Married | <0.001 | 0.002 | (-0.005, 0.005) | 0.992 |
| Political Interest | 0.018 | 0.011 | (-0.003, 0.040) | 0.098 |
| Gender Roles | 0.094 | 0.027 | (0.042, 0.146) | <0.001 |
| Libertarianism-Authoritarianism | 0.023 | 0.013 | (-0.002, 0.048) | 0.068 |
| Left-Right | 0.013 | 0.008 | (-0.003, 0.029) | 0.113 |

*Coefficient component of decomposition for Victim Blaming*

| Variable | Coefficient | *SE* | 95% CI | *p* |
| --- | --- | --- | --- | --- |
| Intercept | -0.719 | 0.800 | (-2.287, 0.850) | 0.369 |
| Gender | 0.061 | 0.075 | (-0.086, 0.207) | 0.417 |
| Age | 0.466 | 1.306 | (-2.093, 3.025) | 0.721 |
| Age Squared | -0.086 | 0.773 | (-1.600, 1.429) | 0.912 |
| Education | -0.124 | 0.226 | (-0.568, 0.319) | 0.583 |
| Occupational Class | -0.078 | 0.145 | (-0.363, 0.207) | 0.593 |
| Religious | -0.046 | 0.058 | (-0.160, 0.068) | 0.431 |
| Married | 0.025 | 0.073 | (-0.119, 0.169) | 0.732 |
| Political Interest | 0.394 | 0.207 | (-0.011, 0.799) | 0.057 |
| Gender Roles | -0.063 | 0.185 | (-0.425, 0.300) | 0.735 |
| Libertarianism-Authoritarianism | 0.280 | 0.346 | (-0.399, 0.958) | 0.419 |
| Left-Right | 0.023 | 0.191 | (-0.351, 0.396) | 0.906 |

*Interaction component of decomposition for Victim Blaming*

| Variable | Coefficient | *SE* | 95% CI | *p* |
| --- | --- | --- | --- | --- |
| Gender | 0.002 | 0.006 | (-0.009, 0.014) | 0.683 |
| Age | -0.018 | 0.0516 | (-0.119, 0.083) | 0.727 |
| Age Squared | 0.006 | 0.055 | (-0.101, 0.113) | 0.912 |
| Education | 0.005 | 0.010 | (-0.015, 0.025) | 0.613 |
| Occupational Class | 0.002 | 0.004 | (-0.007, 0.010) | 0.689 |
| Religious | -0.021 | 0.027 | (-0.074, 0.031) | 0.423 |
| Married | <0.001 | 0.003 | (-0.007, 0.007) | 0.985 |
| Political Interest | -0.020 | 0.013 | (-0.045, 0.005) | 0.109 |
| Gender Roles | -0.010 | 0.031 | (-0.070, 0.050) | 0.745 |
| Libertarianism-Authoritarianism | 0.014 | 0.018 | (-0.022, 0.050) | 0.440 |
| Left-Right | 0.001 | 0.010 | (-0.018, 0.020) | 0.911 |

### **3.2 K-O-B Decomposition: Perpetrator Culpability**

*Endowment component of decomposition for Perpetrator Culpability*

| Variable | Coefficient | *SE* | 95% CI | *p* |
| --- | --- | --- | --- | --- |
| Gender | 0.004 | 0.005 | (-0.005, 0.014) | 0.363 |
| Age | -0.026 | 0.024 | (-0.073, 0.021) | 0.274 |
| Age Squared | 0.042 | 0.026 | (-0.009, 0.093) | 0.107 |
| Education | -0.009 | 0.006 | (-0.020, 0.002) | 0.117 |
| Occupational Class | <0.001 | 0.002 | (-0.004, 0.004) | 0.878 |
| Religious | -0.005 | 0.011 | (-0.027, 0.017) | 0.639 |
| Married | <-0.001 | 0.002 | (-0.004, 0.004) | 0.949 |
| Political Interest | -0.004 | 0.005 | (-0.014, 0.006) | 0.467 |
| Gender Roles | -0.014 | 0.011 | (-0.035, 0.008) | 0.225 |
| Libertarianism-Authoritarianism | -0.061 | 0.014 | (-0.089, 0.034) | <0.001 |
| Left-Right | -0.007 | 0.004 | (-0.015, 0.002) | 0.111 |

*Coefficient Component of Decomposition for Perpetrator Culpability*

| Variable | Coefficient | *SE* | 95% CI | *p* |
| --- | --- | --- | --- | --- |
| Intercept | -0.117 | 0.438 | (-0.975, 0.742) | 0.790 |
| Gender | -0.090 | 0.040 | (-0.168, -0.013) | 0.022 |
| Age | -0.931 | 0.663 | (-2.231, 0.369) | 0.161 |
| Age Squared | 0.506 | 0.369 | (-0.216, 1.229) | 0.170 |
| Education | -0.166 | 0.120 | (-0.402, 0.069) | 0.165 |
| Class | 0.063 | 0.086 | (-0.106, 0.232) | 0.466 |
| Religious | -0.020 | 0.031 | (-0.080, 0.040) | 0.520 |
| Married | -0.003 | 0.042 | (-0.085, 0.078) | 0.934 |
| Political Interest | 0.035 | 0.116 | (-0.193, 0.263) | 0.765 |
| Gender Roles | -0.061 | 0.084 | (-0.225, 0.104) | 0.469 |
| Libertarianism-Authoritarianism | 0.113 | 0.196 | (-0.271, 0.496) | 0.565 |
| Left-Right | 0.291 | 0.116 | (0.064, 0.517) | 0.012 |

*Interaction Component of Decomposition for Perpetrator Culpability*

| Variable | Coefficient | *SE* | 95% CI | *p* |
| --- | --- | --- | --- | --- |
| Gender | -0.004 | 0.005 | (-0.013, 0.005) | 0.420 |
| Age | 0.034 | 0.029 | (-0.023, 0.092) | 0.241 |
| Age Squared | -0.034 | 0.029 | (-0.091, 0.023) | 0.241 |
| Education | 0.007 | 0.006 | (-0.005, 0.019) | 0.273 |
| Class | -0.001 | 0.002 | (-0.006, 0.004) | 0.583 |
| Religious | -0.009 | 0.014 | (-0.038, 0.019) | 0.524 |
| Married | <0.001 | 0.002 | (-0.003, 0.003) | 0.994 |
| Political Interest | -0.002 | 0.006 | (-0.013, 0.010) | 0.772 |
| Gender Roles | -0.010 | 0.014 | (-0.036, 0.017) | 0.472 |
| Libertarianism-Authoritarianism | 0.006 | 0.010 | (-0.014, 0.026) | 0.574 |
| Left-Right | 0.014 | 0.007 | (0.001, 0.027) | 0.036 |

## 4. Models used for moderation analysis

### 4.1 Victim Blaming

#### Political Interest x Year Interaction: Victim Blaming

| *Predictors* | *Estimates* | *SE* | *p* |
| --- | --- | --- | --- |
| Intercept | 1.557 | (0.388) | <0.001 |
| Gender (Woman) | 0.152 | (0.068) | 0.027 |
| Age | -0.079 | (0.012) | <0.001 |
| Age Squared | 0.001 | (<0.001) | <0.001 |
| Education | 0.013 | (0.038) | 0.727 |
| Class | -0.011 | (0.019) | 0.555 |
| Religious Identity (Religious) | 0.192 | (0.070) | 0.006 |
| Marital Status (Married) | 0.008 | (0.068) | 0.906 |
| Political Interest | 0.001 | (0.038) | 0.972 |
| Gender Roles | 0.374 | (0.052) | <0.001 |
| Libertarianism-Authoritarianism | 0.172 | (0.049) | <0.001 |
| Left-Right | 0.123 | (0.043) | 0.004 |
| Survey Year | 0.176 | (0.188) | 0.349 |
| Political Interest x Survey Year | -0.094 | (0.056) | 0.094 |
| Observations | 2136 | | |
| R^2^ / R^2^ adjusted | 0.201 / 0.197 | | |

#### Gender x Year Interaction: Victim Blaming

| *Predictors* | *Estimates* | *SE* | *p* |
| --- | --- | --- | --- |
| Intercept | 1.651 | (0.384) | <0.001 |
| Gender (Woman) | 0.176 | (0.087) | 0.043 |
| Age | -0.079 | (0.012) | <0.001 |
| Age Squared | 0.001 | (<0.001) | <0.001 |
| Education | 0.012 | (0.036) | 0.745 |
| Class | -0.010 | (0.019) | 0.572 |
| Religious Identity (Religious) | 0.201 | (0.070) | 0.004 |
| Marital Status (Married) | 0.005 | (0.068) | 0.947 |
| Political Interest | -0.036 | (0.031) | 0.244 |
| Gender Roles | 0.370 | (0.052) | <0.001 |
| Libertarianism-Authoritarianism | 0.176 | (0.049) | <0.001 |
| Left-Right | 0.122 | (0.043) | 0.005 |
| Survey Year | -0.079 | (0.099) | 0.964 |
| Gender x Survey Year | -0.069 | (0.132) | 0.600 |
| Observations | 2136 | | |
| R^2^ / R^2^ adjusted | 0.200/0.196 | | |

### 4.2 Perpetrator Culpability

#### Left-Right Political Orientation x Year Interaction: Perpetrator Culpability

| *Predictors* | *Estimates* | *SE* | *p* |
| --- | --- | --- | --- |
| Intercept | 4.164 | (0.220) | <0.001 |
| Gender (Woman) | 0.108 | (0.038) | 0.005 |
| Age | 0.003 | (0.006) | 0.697 |
| Age Squared | <0.001 | (<0.001) | 0.113 |
| Education | 0.043 | (0.020) | 0.034 |
| Class | 0.004 | (0.010) | 0.681 |
| Religious Identity (Religious) | -0.058 | (0.039) | 0.136 |
| Marital Status (Married) | 0.057 | (0.038) | 0.134 |
| Political Interest | 0.031 | (0.017) | 0.070 |
| Gender Roles | -0.080 | (0.029) | 0.006 |
| Libertarianism-Authoritarianism | -0.341 | (0.027) | <0.001 |
| Left-Right | 0.063 | (0.032) | 0.047 |
| Survey Year | 0.652 | (0.114) | <0.001 |
| Left-Right x Survey Year | -0.119 | (0.047) | 0.011 |
| Observations | 2133 | | |
| R^2^ / R^2^ adjusted | 0.227 / 0.222 | | |

#### Gender x Year Interaction: Perceived Perpetrator Culpability

| *Predictors* | *Estimates* | *SE* | *p* |
| --- | --- | --- | --- |
| Intercept | 4.320 | (0.214) | <0.001 |
| Gender (Woman) | 0.049 | (0.048) | 0.310 |
| Age | 0.002 | (0.006) | 0.740 |
| Age Squared | <0.001 | (<0.001) | 0.127 |
| Education | 0.042 | (0.020) | 0.035 |
| Class | 0.044 | (0.010) | 0.672 |
| Religious Identity (Religious) | -0.060 | (0.039) | 0.124 |
| Marital Status (Married) | 0.059 | (0.038) | 0.123 |
| Political Interest | 0.030 | (0.017) | 0.075 |
| Gender Roles | -0.081 | (0.029) | 0.005 |
| Libertarianism-Authoritarianism | -0.338 | (0.027) | <0.001 |
| Left-Right | 0.010 | (0.024) | 0.663 |
| Survey Year | 0.298 | (0.055) | <0.001 |
| Gender x Survey Year | 0.149 | (0.074) | 0.043 |
| Observations | 2133 | | |
| R^2^ / R^2^ adjusted | 0.226 / 0.222 | | |

# **References**

Google Trends. (2024, August 10). Sexual assault. Retrieved from Google Trends: <https://trends.google.com/trends/explore?date=2014-01-01%202020-01-01&geo=GB-SCT&q=Sexual%20assault>

Google Trends. (2024, August 10). Sexual harassment. Retrieved from Google Trends: <https://trends.google.com/trends/explore?date=2014-01-01%202020-01-01&geo=GB-SCT&q=Sexual%20harassment>

Google Trends. (2025, April 10). Equally Safe and #MeToo. Retreived from Google Trends: <https://trends.google.com/trends/explore?date=2014-01-01%202020-01-01&geo=GB-SCT&q=Equally%20Safe,%23MeToo&hl=en>

1. A *p >* 0.05 denotes that the observed covariance matrix does not significantly differ from the expected covariance matrix, indicating that the model fits well. [↑](#footnote-ref-1)
